# Simultaneous Quantification of Amino Metabolites in Multiple Metabolic Pathways Using Ultra-High Performance Liquid Chromatography with Tandem-mass Spectrometry

**DOI:** 10.1038/s41598-017-01435-7

**Published:** 2017-05-03

**Authors:** Jin Wang, Lihong Zhou, Hehua Lei, Fuhua Hao, Xin Liu, Yulan Wang, Huiru Tang

**Affiliations:** 1 School of Life Science and Technology, Huazhong University of Science and Technology, Wuhan, 430074 China; 2 State Key Laboratory of Genetic Engineering, Zhongshan Hospital and School of Life Sciences, Fudan University, Shanghai International Centre for Molecular Phenomics, Collaborative Innovation Center for Genetics and Development, Shanghai, 200438 China; 3 CAS Key Laboratory of Magnetic Resonance in Biological Systems, State Key Laboratory of Magnetic Resonance and Atomic and Molecular Physics, National Centre for Magnetic Resonance in Wuhan, Wuhan Institute of Physics and Mathematics, Chinese Academy of Sciences, Wuhan, 430071 China; 4 Collaborative Innovation Center for Diagnosis and Treatment of Infectious Diseases, Zhejiang University, Hangzhou, 310058 China

## Abstract

Metabolites containing amino groups cover multiple pathways and play important roles in redox homeostasis and biosyntheses of proteins, nucleotides and neurotransmitters. Here, we report a new method for simultaneous quantification of 124 such metabolites. This is achieved by derivatization-assisted sensitivity enhancement with 5-aminoisoquinolyl-N-hydroxysuccinimidyl carbamate (5-AIQC) followed with comprehensive analysis using ultra-high performance liquid chromatography and electrospray ionization tandem mass spectrometry (UHPLC-MS/MS). In an one-pot manner, this quantification method enables simultaneous coverage of 20 important metabolic pathways including protein biosynthesis/degradation, biosyntheses of catecholamines, arginine and glutathione, metabolisms of homocysteine, taurine-hypotaurine etc. Compared with the reported ones, this method is capable of simultaneously quantifying thiols, disulfides and other oxidation-prone analytes in a single run and suitable for quantifying aromatic amino metabolites. This method is also much more sensitive for all tested metabolites with LODs well below 50 fmol (at sub-fmol for most tested analytes) and shows good precision for retention time and quantitation with inter-day and intra-day relative standard deviations (RSDs) below 15% and good recovery from renal cancer tissue, rat urine and plasma. The method was further applied to quantify the amino metabolites in silkworm hemolymph from multiple developmental stages showing its applicability in metabolomics and perhaps some clinical chemistry studies.

## Introduction

Metabolism denotes all chemical transformations in living systems and quantifying the metabolite composition (metabonome/metabolome) of such integrated biological systems is vitally important for understanding the molecular basis of such systems. Metabonomics and metabolomics are science for accurate metabonomic (and/or metabolomic) analysis of the dynamic metabolic changes in cells, tissues and whole organisms^1–5^. Therefore, metabonomic/metabolomic analyses have already found widespread applications in revealing the biochemistry details for some basic living processes^6–9^, pathogenesis and progressions^10–12^, systems responses towards xenobiotics^13–17^ and clinical interventions^18–21^, symbiotic interactions in mammals^22–27^ and disease diagnosis and prognosis^28–32^. These analyses ideally require quantification of all metabolites including amino acids, nucleic acids, carboxylic acids, carbohydrates, lipids, and small peptides in complex biological matrices^33^ so as to define the overall metabonomic phenotypes of the studied systems. In practice, however, a single such analysis nowadays can only cover some of all these metabolites due to the diversity of molecular types, matrices, physicochemical properties, dynamic ranges of concentration for these metabolites.

Quantitative analyses of certain targeted metabolomes are often required to obtain accurate and detailed information about some specific metabolites especially in answering biological questions in the hypothesis-driven studies. For this purpose, both gas chromatography-mass spectrometry (GC-MS) and liquid chromatography-mass spectrometry (LC-MS) approaches have been widely employed due to their outstanding metabolite selectivity and sensitivity^34^. Chromatographic separations enable reduction of the sample complexity at detectors alleviating ionization suppression in the subsequent mass spectral acquisitions. Recently developed UHPLC techniques using sub-two μm particles have offered much higher chromatographic resolution and efficiency (or shorter analytical time)^35, 36^ than conventional HPLC. The hyphenated UHPLC and tandem mass spectrometry (UHPLC-MS/MS) with multiple reaction monitoring (MRM) have found widespread applications in quantitative analyses of various sets of specific metabolomes^37–40^ with greatly enhanced throughtput, dynamic range, specificity and sensitivity^35–40^.

Amino group containing metabolites representing an important subset of metabonome cover many important metabolic pathways and possess a variety of vital biological functions. These metabolites include proteinogenic and non-proteinogenic amino acids carrying amino and acidic (e.g., carboxyl or sulfonic) groups, post-translationally modified (methylated, acetylated and phosphorylated) amino acids, aliphatic and aromatic amines, small peptides, catecholamines, thiol and disulfide containing amino metabolites. These metabolites cover dozens of important metabolic pathways and quantitative analysis of them is hence critically important for pathophysiology studies and biomarkers discoveries^41, 42^. Since most of these amino metabolites are fairly hydrophilic, they are often not suitable for straightforward reverse-phase separation and, in theory, can be analyzable with HILIC or ion-pair chromatography^40^. However, these techniques have limited potentials in quantitative metabonomic phenotyping due to their poor chromatographic reproducibility, sensitivity, peak shapes and long equilibration times. Reagents used in ion-pair chromatography also cause undesirable ion suppression effects in the positive ion mode so that a dedicated spectrometer is often required as reported^43^.

Derivatization-based reversed-phase LC-MS analysis is an excellent approach for quantification of amino metabolites especially with efficient amino-group specific tags employed^44^. The traditional tagging reagents include O-phthalaldehyde (OPA)^45^, 9-fluorenylmethylchloroformate (FMOC-Cl)^46^, 5-(dimethylamino)-naphthalene-1-sulfonyl chloride (Dansyl-Cl)^38^, phenylisothiocyanate (PITC)^47^ and 6-aminoquinolyl-N-hydroxysuccinimidyl carbamate (6-AQC)^37, 48^. Amongst them, 6-AQC-based method showed good promising by simultaneously quantifying 46 amino analytes with excellent selectively for both primary and secondary amino groups and suitability for the oxidation-prone analytes (e.g., cysteine, dopamine, N-acetyl-5-hydroxytryptamine) by employing antioxidants (ascorbic acid and TCEP)^37^. However, this method is neither suitable for some important aromatic amino metabolites (such as 3-aminosalicylic acid, 3-hydroxyanthranilic acid, 4-aminobenzoic acids and 4-aminohippuric acid), nor for simultaneous quantification of metabolites containing thiol groups (e.g., cysteine and glutathione) and their corresponding disulfides (cystine and GSSG)^37^ in an “one-pot” manner (in a single run). These thiol- and disulfide-containing metabolites often have to be quantified separately^49–51^ leading to substantial compromise for analytical throughputs with multiple analyses required for different subclasses of amino metabolites. It is also worth-noting that quantities of thiols and disulfides have completely different biological implications. For instance, GSH often plays vital roles in signaling and redox homeostasis and the GSH-to-GSSG ratio is an indicator for oxidative stress^49–51^. For the time being, however, no methods are available for simultaneous quantification of all amino metabolites carrying thiol and disulfide groups concurrently with large number of other amino metabolites in an “one-pot” fashion.

In this work, we report a new derivatization-assisted sensitivity enhancement for quantitative metabolomics method for simultaneous quantification of amino compounds tagged with 5-aminoisoquinolyl-N-hydroxysuccinimidylcarbamate (5-AIQC) using UHPLC-MS/MS techniques. This method showed excellent suitability for quantifying many aromatic amino metabolites which could not be analyzed with the 6-AQC method^37^ and better sensitivity for most metabolites than the 6-AQC-based method^37^. This method enabled simultaneous quantification of multiple subclasses of analytes in a one-pot fashion (in a single run) including both the thiol- and disulfide-containing metabolites, amino acids, biogenic amines, small peptides and monoamine neurotransmitters. This new method further showed excellent applicability in quantitative analysis of amino metabolites in different matrices including rat urine and plasma, human kidney tissue and silkworm hemolymph.

## Results and Discussion

### Derivatization of amino analytes by 5-aminoisoquinolyl-N-hydroxysuccinimidylcarbamate(5-AIQC)

**Figure 1:**
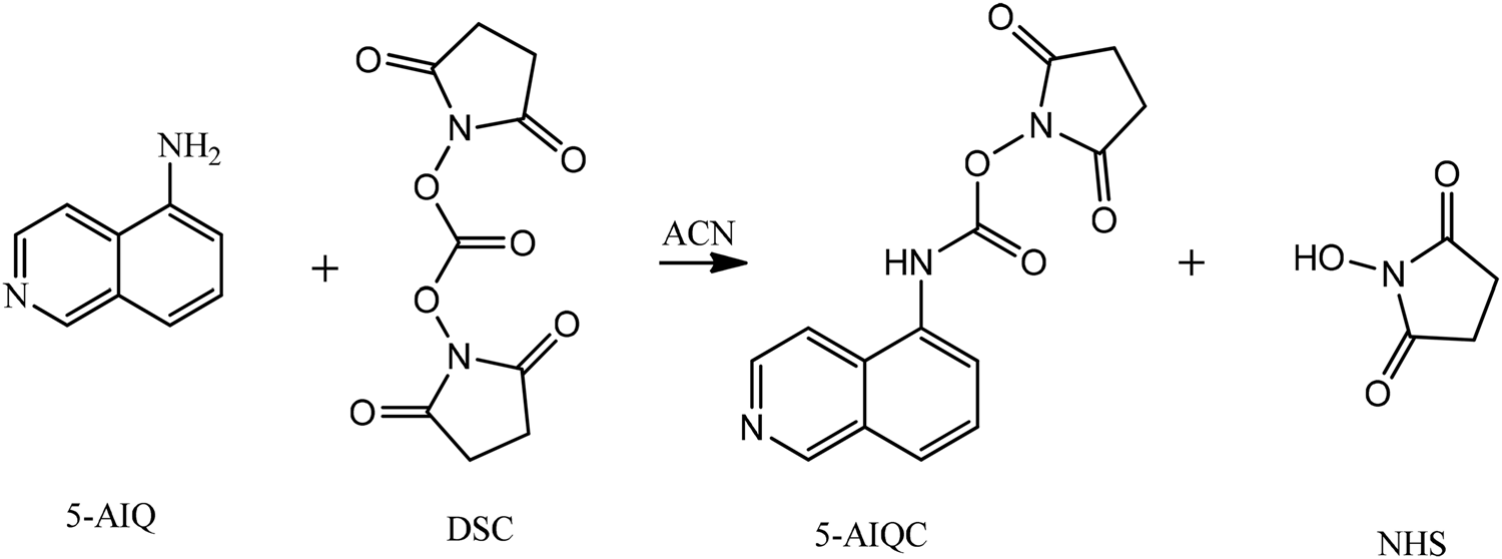
Synthesis of 5-aminoisoquinolyl-N-hydroxysuccinimidyl carbamate (5-AIQC).

**Figure 2:**
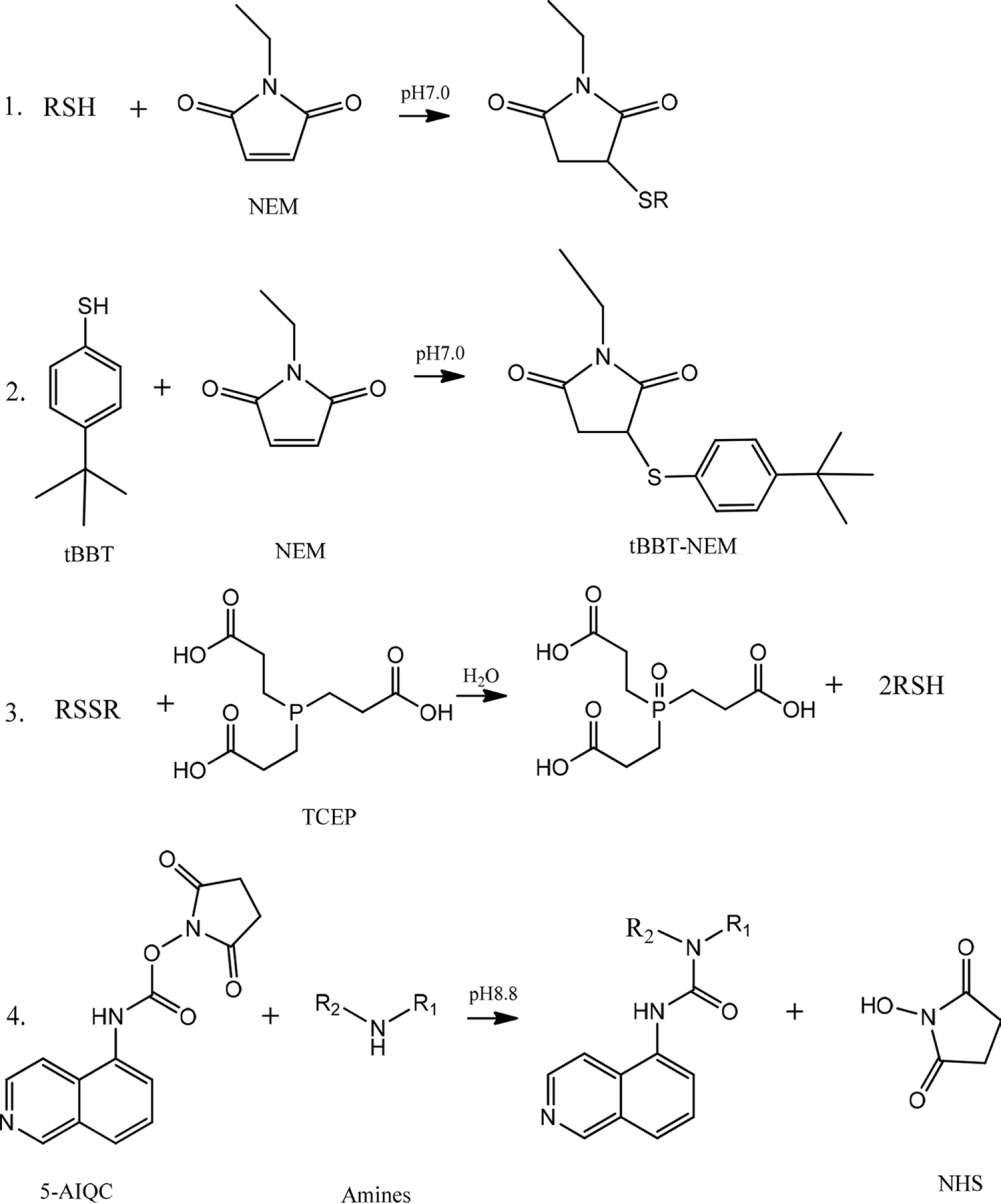
Schemes for 5-AIQC derivatization of amino analytes with thiol and disulfide groups in one pot.

5-AIQC was readily prepared at ambient temperature by simply adding 5-aminoisoquinoline to excess N,N′-disuccinimidylcarbonate (Fig. 1). 5-AIQC rapidly reacts with both the primary and secondary amino groups of analytes (within 10 mins) at the ambient temperature with excellent selectivity producing asymmetric ureas (Fig. 2, Supplementary Fig. S1) which are stable at room temperature. Although 5-AIQC also reacts with phenolic hydroxyl groups (e.g., in tyrosine), mild heating (55 °C) easily facilitates degradation of such adducts leaving only the amino-5-AIQC adducts intact as in the case of 6-AQC^37, 48^. So far, 5-AIQC has not been employed for analysis of amino compounds using mass spectrometry to the best of our knowledge though synthesis of 5-AIQC was reported in 1991 as a potential fluorescent tag for amino acids^52^. With higher pKa for isoquinoline than quinoline, 5-AIQC derivatized amino compounds is expected to have better sensitivities in the positive ion mass spectrometry than 6-AQC-adducts, which will be discussed later.

To make the analytical method applicable for the oxidation-prone metabolites including thiols and catecholamines in biological samples, addition of antioxidants including TCEP and ascorbic acid is necessary^37^. However, TCEP will convert disulfides into thiols^37^. To enable simultaneous quantification of the amino-containing thiols (such as cysteine and GSH) and disulfides (such as cystine and glutathione disulfide), we employed N-ethylmaleimide (NEM) here to trap thiols through click reaction forming RSH-NEM adducts (Fig. 2, Supplementary Fig. S1) which completed within 2 min at pH7.0 avoiding very slow reactions of NEM with amino group^50, 53^. The remaining NEM was then quenched by click reaction with excess 4-*tert*-butylbenzenethiol (tBBT) to form stable NEM-tBBT adduct. After trapping of original thiols, 20 mM TCEP solution in borate buffer (200 mM, pH 8.8) containing 1 mM ascorbic acid was added to convert all disulfides into thiols to avoid multiple tagging and to prevent oxidation-prone metabolites (e.g., dopamine, tryptamine and norepinephrine) together with the TCEP-generated thiols from oxidization during analysis^37^. Therefore, the newly produced thiols from disulfides (RSSR) can be readily distinguished from the original thiols during quantification. After these treatments, the amino compounds were then easily quantified as 5-AIQC adducts and the thiol-containing ones were quantified as 5-AIQC-RSH-NEM adducts but disulfide-containing ones as 5-AIQC-RSH adducts in an one-pot manner. Since both tBBT and its stable NEM-tBBT adduct formed from quenching NEM are much more hydrophobic than all 5-AIQC-derivatised compounds, these two by-products can be conveniently flushed into waste with 95% CH_3_OH after elution of all the 5-AIQC-analyte adducts. Although NEM can be hydrolyzed slowly at pH8.8 to open its ring, such hydrolysis was minimal within the total period required for derivatization (less than 15 mins) and LC-MS analysis. For instance, we found that only less than 3% 5-AIQC-NEM-Cys was hydrolyzed in this study (Supplementary Fig. S2). With good NEM stability under acidic condition (pH < 5.0)^54^, sufficient formic acid was added immediately after derivatization to lower pH to about 2.5. Under such acidic condition, the thiol-NEM adducts were all very stable within 48 h without extra hydrolysis detectable (Supplementary Fig. S2) and a 20-fold excess of 5-AIQC against the total amino groups was sufficient to complete derivatization of all analytes (Supplementary Fig. S3). Interestingly, the 5-AIQC derivatives of GSH, homocysteine, γ-Glu-Cys, DL-2,6-diaminopimelic acid and DL-lanthionine (i.e., GSH-NEM-AIQC, Hcys-NEM-AIQC, γ-Glu-Cys-NEM-AIQC, DL-2,6-diaminopimelate-AIQC_2_ and DL-lanthionine-AIQC_2_) all showed two chromatographic peaks (Supplementary Fig. S4) probably due to the presence of two different ionization forms (for their carboxyl groups) at the given pH of mobile phase. This is further supported by the observable alterations of these peaks and only a single chromatographic peak for DL-2,6-diaminopimelate-AIQC_2_ with an increase of elution solvent acidity (to pH~2.36). Nevertheless, they all showed excellent sensitivity, linearity, retention time precision and peak shapes taking both these peaks into considerations (Table 1). However, 5-AIQC failed in tagging adenine, amide, guanido groups as in the case of 6-AQC^37^.

**Table 1:** Data for 126 amino analytes in the form of their 5-AIQC-adducts including the neutral formula, theoretical m/z, precursor (Q1) and fragment (Q3) ions, retention time (RT), collision energy (CE), fragmentor voltage (FV), linear range and coefficients (R^2^) and the on-column limits of detection for the 5-AIQC-adducts (LOD^a^) and 6-AQC-adducts (LOD^b^) from the same method.

NO.	Analytes	Neutral formula	5-AIQC-adduct m/z	Q1 (m/z)	Q3 (m/z)	FV	CE	RT	LOD^a^ (fmol)	LOD^b^ (fmol)	Linear range (μM)	R^2^	Categories
1	D-Mannosamine	C_16_H_19_N_3_O_6_	350.1347	350	171	140	40	1.328	17.3	80.6	5.8–500	0.9978	AS
2	D-(+)-Glucosamine	C_16_H_19_N_3_O_6_	350.1347	350	171	140	40	1.498	9.4	61.2	3.1–500	0.9941	AS
3	D-(+)-Galactosamine	C_16_H_19_N_3_O_6_	350.1347	350	171	140	40	1.892	15.4	113.6	5.1–500	0.9958	AS
4	1-Deoxynojirimycin	C_16_H_19_N_3_O_5_	334.1397	334	171	120	20	3.347	0.1	0.2	0.02–200	0.9977	AS
5	L-Asparagine	C_14_H_14_N_4_O_4_	303.1088	303	171	120	10	3.847	1.3	2.2	0.4–200	0.9986	PAA
6	L-Histidine	C_16_H_15_N_5_O_3_	326.1248	326	171	100	20	3.957	1.8	5.5	0.6–200	0.9997	PAA
7	L-Serine	C_13_H_13_N_3_O_4_	276.0979	276	171	100	10	4.024	0.5	0.9	0.2–200	0.9986	PAA
8	Glycine	C_12_H_11_N_3_O_3_	246.0873	246	171	100	10	4.278	0.6	0.9	0.2–200	0.9998	PAA, NT
9	L-Glutamine	C_15_H_16_N_4_O_4_	317.1244	317	171	120	10	4.74	2.9	6.7	1–200	0.9998	PAA
10	L-Arginine	C_16_H_20_N_6_O_3_	345.167	345	171	120	40	5.016	15.0	30.0	5–200	0.9992	PAA
11	L-Aspartic acid	C_14_H_13_N_3_O_5_	304.0928	304	171	120	20	5.021	1.4	5.5	0.5–200	0.9998	PAA, NT
12	L-Glutamic acid	C_15_H_15_N_3_O_5_	318.1084	318	171	120	10	5.703	1.2	3.2	0.4–200	0.9999	PAA, NT
13	L-Threonine	C_14_H_15_N_3_O_4_	290.1135	290	171	100	20	5.979	0.7	2.3	0.2–200	0.9987	PAA
14	L-Alanine	C_13_H_13_N_3_O_3_	260.103	260	171	100	20	6.27	0.3	0.3	0.1–200	0.999	PAA
15	L-Proline	C_15_H_15_N_3_O_3_	286.1186	286	171	100	20	6.538	0.5	7.0	0.2–200	0.996	PAA
16	L-Tyrosine	C_19_H_17_N_3_O_4_	352.1292	352	171	120	10	8.709	0.4	0.5	0.1–100	0.999	PAA
17	L-Methionine	C_15_H_17_N_3_O_3_S	320.1063	320	171	120	20	8.911	1.1	1.9	0.4–200	0.9971	PAA, S-AA
18	L-Lysine	C_26_H_26_N_6_O_4_	244.1081	244	171	100	20	9.202	0.6	1.3	0.2–200	0.9997	PAA
19	L-Valine	C_15_H_17_N_3_O_3_	288.1343	288	171	120	20	9.515	0.8	1.5	0.3–200	0.9975	PAA
20	L-Isoleucine	C_16_H_19_N_3_O_3_	302.1499	302	171	120	20	12.671	1.8	2.4	0.6–200	0.9988	PAA
21	L-Leucine	C_16_H_19_N_3_O_3_	302.1499	302	171	120	20	12.738	1.6	2.7	0.5–200	0.9955	PAA
22	DL-Phenylalanine	C_19_H_17_N_3_O_3_	336.1343	336	171	120	20	12.783	0.5	0.5	0.2–200	0.9998	PAA
23	L-Tryptophan	C_21_H_18_N_4_O_3_	375.1452	375	171	140	20	12.992	1.0	1.0	0.3–200	0.9985	PAA
24	D-Homoserine	C_14_H_15_N_3_O_4_	290.1135	290	171	100	20	4.942	0.9	2.5	0.3–200	0.9997	N-PAA
25	Saccharopine	C_21_H_26_N_4_O_7_	447.1874	447	171	120	20	5.518	4.2	15.8	1.4–280	0.9986	N-PAA
26	Argininosuccinic acid	C_20_H_24_N_6_O_7_	461.1779	461	171	160	40	5.561	7.7	79.4	2.6–500	0.9726	N-PAA, MAA
27	β-alanine	C_13_H_13_N_3_O_3_	260.103	260	171	100	10	5.844	0.3	0.4	0.1–200	0.9996	N-PAA
28	L-Citrulline	C_16_H_19_N_5_O_4_	346.151	346	171	120	20	5.867	2.1	2.9	0.7–200	0.9996	N-PAA
29	L-Homoarginine	C_17_H_22_N_6_O_3_	359.1826	359	171	120	20	5.934	8.9	26.7	3–100	0.9997	N-PAA
30	γ-Aminobutyric acid	C_14_H_15_N_3_O_3_	274.1186	274	171	120	20	6.665	1.2	2.7	0.4–200	0.9983	N-PAA, NT
31	L-Homocitrulline	C_17_H_21_N_5_O_4_	360.1666	360	171	100	20	6.946	1.5	5.8	0.5–200	0.9985	N-PAA
32	L-2-aminoadipic acid	C_16_H_17_N_3_O_5_	332.1241	332	171	120	20	7.075	2.5	4.2	0.8–100	0.9998	N-PAA
33	DL-3-Aminoisobutyric acid	C_14_H_15_N_3_O_3_	274.1186	274	171	120	20	7.172	1.1	2.1	0.4–200	0.9997	N-PAA
34	2-Aminoisobutyric acid	C_14_H_15_N_3_O_3_	274.1186	274	171	100	20	7.613	2.6	8.6	0.9–200	0.991	N-PAA
35	5-Aminovaleric acid	C_15_H_17_N_3_O_3_	288.1343	288	171	120	20	7.754	2.2	5.9	0.7–200	0.9992	N-PAA
36	L-2-Aminobutyric acid	C_14_H_15_N_3_O_2_	274.1186	274	171	100	20	7.792	0.8	1.5	0.3–200	0.9995	N-PAA
37	2,4-diaminobutanoic acid	C_24_H_22_N_6_O_4_	230.0924	230	171	80	20	7.859	3.2	8.3	1.1–200	0.9988	N-PAA
38	DL-2,6-Diaminopimelic acid	C_27_H_26_N_6_O_6_	266.103	266	171	100	20	8.084, 8.208	2.5	7.0	0.8–200	0.9996	N-PAA
39	L-Ornithine	C_25_H_24_N_6_O_4_	237.1002	237	171	100	10	8.321	0.9	1.8	0.3–200	0.9966	N-PAA
40	6-Aminocaproic acid	C_16_H_19_N_3_O_3_	302.1499	302	171	120	20	9.053	2.1	6.3	0.7–200	0.9993	N-PAA
41	3-hydroxykynurenine	C_20_H_18_N_4_O_5_	395.135	395	171	110	20	9.479	3.1	13.2	1.0–200	0.9914	N-PAA
42	L-Norvaline	C_15_H_17_N_3_O_3_	288.1343	288	171	120	20	9.709	0.8	1.7	0.3–200	0.9994	N-PAA
43	D–(−)-α-Phenylglycine	C_18_H_15_N_3_O_3_	322.1186	322	171	100	20	10.224	2.4	3.2	0.8–200	0.9983	N-PAA
44	L-Pipecolic acid	C_16_H_17_N_3_O_3_	300.1343	300	171	100	20	10.336	22.0	249.6	7.3–500	0.9962	N-PAA
45	L-Kynurenine	C_20_H_18_N_4_O_4_	379.1401	379	171	100	20	11.753	1.9	3.2	0.6–100	0.9997	N-PAA
46	L-Norleucine	C_16_H_19_N_3_O_3_	302.1499	302	171	120	20	13.156	2.0	2.2	0.7–100	0.9998	N-PAA
47	Histamine	C_15_H_15_N_5_O	282.1349	282	171	100	20	4.237	2.9	4.4	1.0–500	0.9928	NT, ALA
48	(−)-Norepinephrine	C_18_H_17_N_3_O_4_	340.1292	340	171	120	20	6.882	2.7	4.4	0.9–200	0.9991	NT, ALA
49	(±)-Octopamine	C_18_H_17_N_3_O_3_	324.1343	324	171	100	20	7.59	1.3	1.5	0.4–200	0.9994	NT, ALA
50	Dopamine	C_18_H_17_N_3_O_3_	324.1343	324	171	100	20	8.336	1.4	2.0	0.5–200	0.9988	NT, ALA
51	Serotonin	C_20_H_18_N_4_O_2_	347.1503	347	171	100	20	8.606	1.6	14.8	0.5–500	0.9926	NT, ALA
52	Tyramine	C_18_H_17_N_3_O_2_	308.1394	308	171	120	20	9.358	2.3	4.7	0.8–200	0.9993	NT, ALA
53	3-Methoxytyramine	C_19_H_19_N_3_O_3_	338.1499	338	171	100	20	10.008	0.8	1.3	0.3–50	0.9998	NT, ALA
54	Tryptamine	C_20_H_18_N_4_O	331.1553	331	171	120	20	13.305	3.2	4.6	1.1–200	0.9984	NT, ALA
55	4-Aminophenol	C_16_H_13_N_3_O_2_	280.1081	280	171	100	20	8.165	2.9	50.6	1–200	0.9978	ARA
56	3-hydroxyanthranilic acid	C_17_H_13_N_3_O_4_	324.0979	324	171	100	20	8.866	19.0	—	6.3–100	0.9962	ARA, N-PAA
57	*4*-aminohippuric acid	C_19_H_16_N_4_O_4_	365.1244	365	171	120	20	9.202	30.0	—	10–200	0.9995	ARA, N-PAA
58	Procaine	C_23_H_26_N_4_O_3_	407.2078	407	171	140	20	9.612	174.4	—	58.1–200	0.9899	ARA
59	5-Hydroxyindoleacetic acid	C_20_H_15_N_3_O_4_	362.1135	362	171	100	20	11.373	16.8	105.0	5.6–500	0.973	ARA
60	3-Aminobenzoic acid	C_17_H_13_N_3_O_3_	308.103	308	171	120	10	11.477	4.1	—	1.4–200	0.9939	ARA, N-PAA
61	3-Aminosalicylic acid	C_17_H_13_N_3_O_4_	324.0979	324	171	120	20	11.574	8.7	—	2.9–100	0.9984	ARA, N-PAA
62	4-Aminobenzoic acid	C_17_H_13_N_3_O_3_	308.103	308	171	120	10	11.649	1.7	—	0.6–200	0.9989	ARA, N-PAA
63	N-acetyl-5-hydroxytryptamine	C_22_H_20_N_4_O_3_	389.1608	389	171	120	20	11.761	10.1	62.0	3.4–200	0.9997	ARA
64	L-Cysteic acid	C_13_H_13_N_3_O_6_S	340.0598	340	171	120	20	3.473	5.8	8.7	1.9–200	0.999	S-AA, N-PAA
65	Taurine	C_12_H_13_N_3_O_4_S	296.07	296	171	100	10	4.733	2.5	7.4	0.8–200	0.9998	S-AA, N-PAA
66	Hypotaurine	C_12_H_13_N_3_O_3_S	280.075	280	171	120	10	4.875	0.8	1.1	0.3–100	0.9995	S-AA, N-PAA
67	S-(5’-Adenosyl)-L-methionine	C_25_H_28_N_8_O_6_S	569.1925	569	171	140	20	5.146	19.5	36.3	6.5–200	0.9973	S-AA, N-PAA
68	DL-Methionine sulfone	C_15_H_17_N_3_O_6_S	352.0962	352	171	100	10	5.546	0.9	3.1	0.3–200	0.9997	S-AA, N-PAA
69	DL-methionine sulfoxide	C_15_H_17_N_3_O_4_S	336.1013	336	171	100	20	5.606	2.0	7.1	0.7–200	0.9998	S-AA, N-PAA
70	Glutathione disulfide	C_20_H_23_N_5_O_7_S	478.1391	478	171	160	20	6.844	3.6	4.3	1.2–40	0.9924	S-AA, SP
71	L-Cystine	C_13_H_14_N_3_O_3_S	292.075	292	171	120	20	6.882	1.2	1.6	0.4–50	0.9917	S-AA, N-PAA
72	Cystamine	C_12_H_13_N_3_OS	248.0852	248	171	100	10	7.232	2.9	3.5	1–100	0.998	S-AA, ALA
73	L-Homocystine	C_14_H_15_N_3_O_3_S	306.0907	306	171	100	20	7.896	2.7	2.8	0.9–50	0.9989	S-AA, N-PAA
74	S-(5’-adenosyl)-L-homocysteine	C_24_H_26_N_8_O_6_S	555.1769	555	171	160	20	8.344	19.0	52.3	6.3–120	0.9862	S-AA, N-PAA
75	Cystathionine	C_27_H_26_N_6_O_6_S	282.089	282	171	100	10	8.956	2.8	8.2	0.9–100	0.9992	S-AA, N-PAA
76	S-(2-Aminoethyl)-L-cysteine	C_25_H_24_N_6_O_4_S	253.0863	253	171	80	10	8.985	1.3	3.0	0.4–200	0.9996	S-AA
77	L-Cysteine	C_19_H_20_N_4_O_5_S	417.1227	417	171	140	20	9.261	3.6	4.0	1.2–100	0.9934	PAA, S-AA
78	Djenkolic Acid	C_27_H_26_N_6_O_6_S_2_	298.075	298	171	100	20	10.112	2.2	2.1	0.7–200	0.9991	S-AA
79	Cysteamine	C_18_H_20_N_4_O_3_S	373.1329	373	171	120	20	10.246	4.1	4.0	1.4–100	0.9943	S-AA, ALA
80	DL-Ethionine	C_16_H_19_N_3_O_3_S	334.122	334	171	100	20	11.201	1.4	4.0	0.5–100	0.9996	S-AA, N-PAA
81	DL-Homocysteine	C_20_H_22_N_4_O_5_S	431.1384	431	171	130	20	10.291, 10.611	6.9	5.9	2.3–100	0.9912	S-AA, N-PAA
82	DL-Lanthionine	C_26_H_24_N_6_O_6_S	275.0812	275	171	100	10	7.986, 8.217	3.1	8.3	1.0–400	0.9986	S-AA, N-PAA
83	Glutathione	C_26_H_30_N_6_O_9_S	603.1868	603	171	120	20	8.784, 9.053	4.1	6.8	1.4–100	0.9995	SP, S-AA
84	γ-Glu-Cys	C_24_H_27_N_5_O_8_S	546.1653	546	171	140	20	8.791, 8.933	0.9	2.3	0.3–250	0.9916	SP, S-AA
85	L-Carnosine (β-ala-L-his)	C_19_H_20_N_6_O_4_	397.1619	397	171	120	20	4.964	18.3	28.4	6.1–200	0.9989	SP
86	L-Anserine (β-ala-N-methyl-his)	C_20_H_22_N_6_O_4_	411.1775	411	171	140	40	5.203	2.8	5.7	0.9–200	0.9935	SP
87	Ala-leu	C_19_H_24_N_4_O_4_	373.187	373	171	130	20	11.574	0.7	1.7	0.2–200	0.9998	SP
88	Ala-Trp	C_24_H_23_N_5_O_4_	446.1823	446	171	140	20	11.753	1.6	5.3	0.5–50	0.9998	SP
89	Leu-Pro	C_21_H_26_N_4_O_4_	399.2027	399	171	130	20	13.678	0.3	0.8	0.1–500	0.9967	SP
90	*trans*-4-Hydroxy-L-proline	C_15_H_15_N_3_O_4_	302.1135	302	171	120	20	3.091	2.2	2.1	0.7–200	0.999	N-PAA, MAA
91	O-Phospho-L-serine	C_13_H_14_N_3_O_7_P	356.0642	356	171	100	10	3.633	23.4	65.2	7.8–1000	0.999	N-PAA, MAA
92	*O*-Phosphorylethanolamine	C_12_H_14_N_3_O_5_P	312.0744	312	171	100	10	3.875	1.2	4.3	0.4–200	0.9993	N-PAA, MAA
93	Sarcosine	C_13_H_13_N_3_O_3_	260.103	260	171	100	10	4.412	4.1	6.2	1.4–200	0.9947	N-PAA, MAA
94	3-Methyl-L-histidine	C_17_H_17_N_5_O_3_	340.1404	340	171	120	20	4.457	7.5	11.4	2.5–100	0.9998	N-PAA, MAA
95	Nε,Nε,Nε-Trimethyllysine	C_19_H_26_N_4_O_3_	180.1075	180	171	120	20	4.524	1.0	3.6	0.3–500	0.9919	N-PAA, MAA
96	O-Phospho-L-threonine	C_14_H_16_N_3_O_7_P	370.0799	370	171	120	20	4.817	3.8	20.7	1.3–1000	0.9904	N-PAA, MAA
97	1-Methyl-L-histidine	C_17_H_17_N_5_O_3_	340.1404	340	171	100	20	4.897	9.4	19.4	3.1–100	0.9995	N-PAA, MAA
98	Asymmetric dimethylarginine	C_18_H_24_N_6_O_3_	373.1983	373	171	120	20	6.24	31.3	37.5	10.4–50	0.9996	N-PAA, MAA
99	O-acetyl-L-serine	C_15_H_15_N_3_O_5_	318.1084	318	171	120	10	6.941	1.9	8.6	0.6–200	0.9995	N-PAA, MAA
100	O-phospho-L-tyrosine	C_19_H_18_N_3_O_7_P	432.0955	432	171	120	20	7.179	17.8	85.2	5.9–1000	0.9932	N-PAA, MAA
101	Nα-Acetyl-L-lysine	C_18_H_22_N_4_O_4_	359.1714	359	171	120	20	7.59	2.0	3.7	0.7–200	0.9996	N-PAA, MAA
102	DL-5-Hydroxylysine	C_26_H_26_N_6_O_5_	252.1055	252	171	100	10	7.986	1.7	3.2	0.6–100	0.9992	N-PAA, MAA
103	5-Hydroxy-L-tryptophan	C_21_H_18_N_4_O_4_	391.1401	391	171	100	20	8.001	2.6	2.5	0.9–200	0.9958	N-PAA, MAA
104	4-Hydroxy-L-isoleucine	C_16_H_19_N_3_O_4_	318.1448	318	171	80	20	8.404	5.2	5.2	1.7–50	0.9998	N-PAA, MAA
105	Ethanolamine	C_12_H_13_N_3_O_2_	232.1081	232	171	100	20	5.024	1.0	5.6	0.3–200	0.9998	ALA
106	Methylamine	C_11_H_11_N_3_O	202.0975	202	171	80	10	5.076	0.6	1.9	0.2–200	0.999	ALA
107	Agmatine	C_15_H_20_N_6_O	301.1771	301	171	120	20	5.68	1.8	6.3	0.6–200	0.9992	NT, ALA
108	Ethylamine	C_12_H_13_N_3_O	216.1131	216	171	100	20	6.613	1.7	20.1	0.6–200	0.9997	ALA
109	Putrescine	C_24_H_24_N_6_O_2_	215.1053	215	171	120	20	9.008	2.5	11.3	0.8–200	0.9998	ALA
110	Cadaverine	C_25_H_26_N_6_O_2_	222.1131	222	171	100	20	10.284	2.5	6.5	0.8–200	0.9996	ALA
111	Spermidine	C_37_H_37_N_9_O_3_	219.4413	219	171	80	20	12.641	3.5	19.9	1.2–500	0.9945	ALA
112	Spermine	C_50_H_50_N_12_O_4_	442.2112	442	171	140	20	13.292	8.1	40.0	2.7–200	0.9959	ALA
113	NH_4_Cl	C_10_H_9_N_3_O	188.0818	188	171	80	10	3.076	0.4	0.7	0.1–200	0.9979	Ammonium
114	Prolinamide	C_15_H_16_N_4_O_2_	285.1346	285	171	80	20	5.501	1.2	3.3	0.4–200	0.9993	ALA
115	Allantoin	C_14_H_12_N_6_O_4_	329.0993	329	171	120	20	6.521	15.8	25.9	5.3–100	0.9989	ALA
116	5-Hydroxydopamine	C_18_H_17_N_3_O_4_	340.1292	340	171	120	20	7.061	3.6	6.1	1.2–50	0.9998	ALA
117	3,4-dihydroxy-DL-phenylalanine	C_19_H_17_N_3_O_5_	368.1241	368	171	120	20	7.725	2.7	3.1	0.9–200	0.9996	ALA
118	DL-Normetanephrine	C_19_H_19_N_3_O_4_	354.1448	354	171	120	20	8.045	2.5	3.2	0.8–100	0.9997	ALA
119	2-Amino-2-methyl-1-propanol	C_14_H_17_N_3_O_2_	260.1394	260	171	100	10	8.091	0.2	0.3	0.1–400	0.9992	ALA
120	1,3-Diaminopropane	C_23_H_22_N_6_O_2_	208.0975	208	171	80	10	8.418	1.8	7.4	0.6–200	0.9993	ALA
121	1,2-Diaminopropane	C_23_H_22_N_6_O_2_	208.0975	208	171	80	10	8.821	0.9	4.4	0.3–200	0.9992	ALA
122	L-Tryptophanamide	C_21_H_19_N_5_O_2_	374.1612	374	171	100	20	10.649	1.1	3.2	0.4–100	0.9991	ALA
123	Isopentylamine	C_15_H_19_N_3_O	258.1601	258	171	120	20	13.382	0.9	2.7	0.3–500	0.9957	ALA
124	Desipramine	C_28_H_28_N_4_O	437.2336	437	171	130	20	14.072	0.3	0.4	0.1–100	0.9995	ALA
125	Methylguanidine	C_22_H_19_N_7_O_2_	207.5873	—	—	—	—	—	—	—	—	—	guanidines
126	Adenosine	C_20_H_19_N_7_O_5_	438.152	—	—	—	—	—	—	—	—	—	NT

AS: amino-saccharides; PAA: proteinogenic amino acids; NT: neurotransmitters; N-PAA: Non-proteinogenic amino acids; ALA: aliphatic amines; ARA: aromatic amines; S-AA: sulfur-containing analytes; SP: small peptides; MAA: modified amino acids.

### UHPLC-ESI-MS/MS Analysis of Amino Compounds

Asymmetric ureas formed from 5-AIQC and all amino metabolites were readily detectable in the positive ion mass spectrometry with MRM mode by showing a common fragment ion at m/z 171 derived from the amino isoquinoline moiety (Table 1). Such derivatized amino analytes have higher hydrophobicity than analytes themselves making reverse-phase UHPLC-MS/MS suitable technique for more sensitive quantitative analysis. We developed an UHPLC-MS/MS method for simultaneous quantification of 124 amino compounds with all parameters systematically optimized for UHPLC (including columns and temperature, mobile phases and gradients, buffers, flow rate and injection volume) and mass spectrometry. With these optimized parameters, both excess by-products, 5-AIQ and NEM-tBBT, were eluted either at the beginning (in the case of 5-AIQ) or end of chromatography and discarded to avoid contaminating source. This new method demonstrated easy coverage of 124 analytes in this work representing 4 amino-saccharides, 20 proteinogenic amino acids, 57 non-proteinogenic amino acids, 17 modified amino acids, 26 aliphatic and 8 aromatic amines, 22 sulfur-containing compounds, 14 monoamine neurotransmitters and 8 small peptides (Table 1, Supplementary Fig. S5).

**Figure 3:**
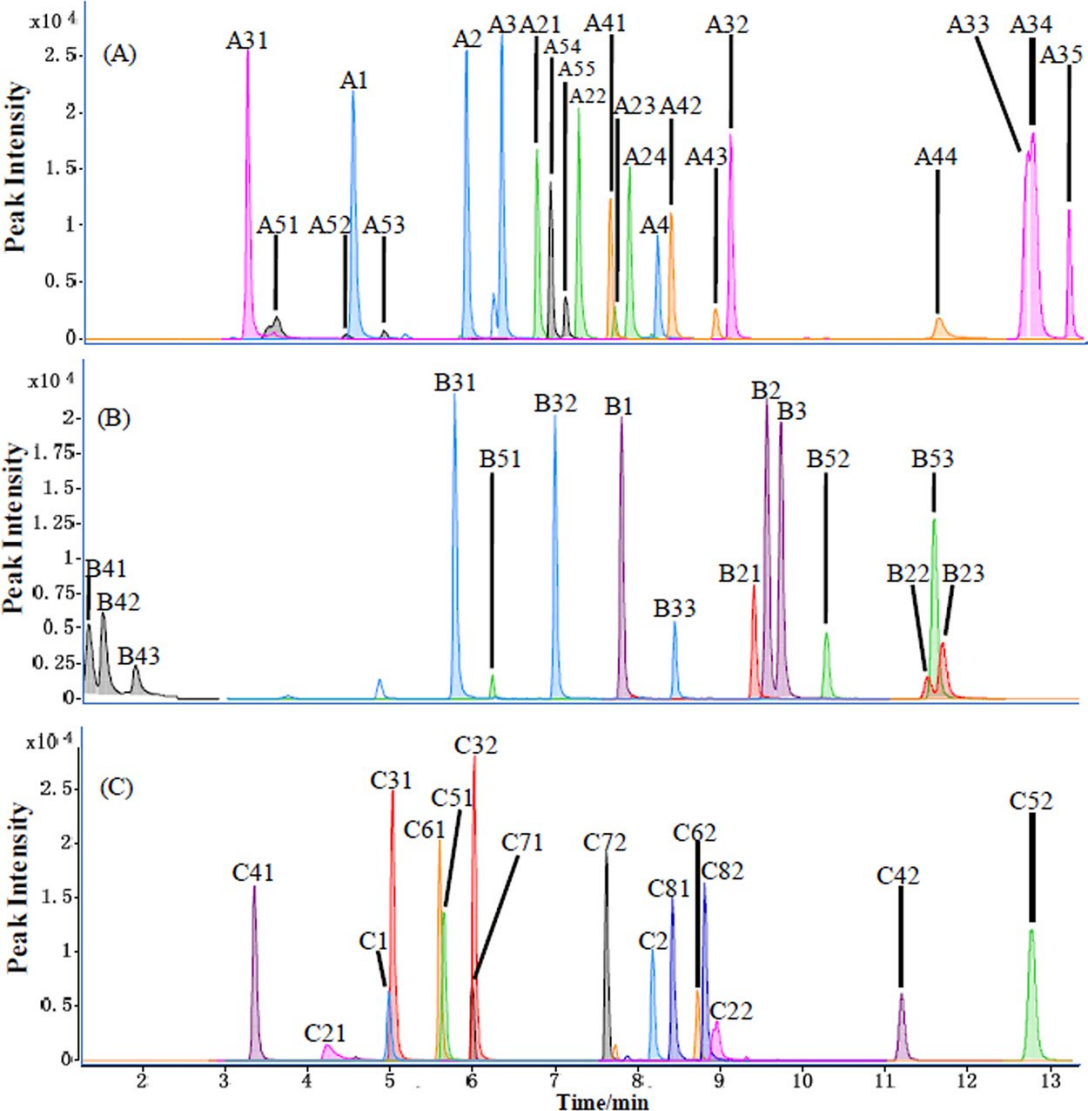
UHPLC-MS/MS chromatograms for some sets of the 5-AIQC-tagged amino analytes having the same pseudomolecular ions (m/z at unit resolution). (**A**) ion m/z 260 (A1: sarcosine; A2: β-alanine; A3: L-alanine; A4: 2-amino-2-methyl-1-propanol); ion m/z 274 (A21: γ-aminobutyric acid; A22: DL-3-aminoisobutyric acid; A23: 2-aminoisobutyric acid; A24: L-2-aminobutyric acid); ion m/z 302 (A31: *trans*-4-hydroxy-L-proline; A32: 6-aminocaproic acid; A33: L-isoleucine; A34: L-leucine; A35: L-norleucine); ion m/z 324 (A41: (±)-octopamine; A42: dopamine; A43: 3-hydroxyanthranilic acid; A44: 3-aminosalicylic acid); ion m/z 340 (A51: L-cysteic acid; A52: 3-methyl-L-histidine; A53: 1-methyl-L-histidine; A54: (−)-norepinephrine; A55: 5-hydroxydopamine). (**B**) ion m/z 288 (B1: 5-aminovaleric acid; B2: L-valine; B3: L-norvaline); ion m/z 308 (B21: tyramine; B22: 3-aminobenzoic acid; B23: 4-aminobenzoic acid); ion m/z 318 (B31: L-glutamic acid; B32: o-acetyl-L-serine; B33: 4-hydroxy-L-isoleucine); ion m/z 350 (B41: D-mannosamine; B42: D-(+)-glucosamine; B43: D-(+)-galactosamine); ion m/z 373 (B51: asymmetric dimethylarginine; B52: cysteamine; B53: Ala-Leu). (**C**) ion m/z 280 (C1: hypotaurine; C2: 4-aminophenol); ion m/z 282 (C21: histamine; C22: cystathionine); ion m/z 290 (C31: D-homoserine; C32: L-threonine); ion m/z 334 (C41: 1-deoxynojirimycin; C42: DL-ethionine); ion m/z 336 (C51: DL-methionine sulfoxide; C52: DL-phenylalanine); ion m/z 352 (C61: DL-methionine sulfone; C62: L-tyrosine); ion m/z 359 (C71: L-homoarginine; C72: Nα-acetyl-L-lysine); ion m/z 208 (C81: 1,3-diaminopropane; C82: 1,2-diaminopropane).

This optimized method enabled many sets of isomers to be chromatographically separated (Fig. 3) making them readily quantifiable. For intance, 5-AIQC derivatives of 5 leucine isomers (isoluecine, leucine, norleucine, hydroxyproline and 6-aminocaproic acid) were separated on column though they all had the same ion m/z 302 (Fig. 3A). 5-AIQC adducts of five three-metabolite sets were readily separated on column and quantified (Fig. 3B), respectively, such as 3 valine isomers with ion m/z 288 (5-aminovaleric acid, L-valine, L-norvaline) and 3 aromatic metabolites with ion m/z 308 (tyramine, 3-aminobenzoic acid, 4-aminobenzoic acid), etc. In the same manner, eight pairs of the 5-AIQC derivatives of analytes were separated and simultaneously quantified (Fig. 3C), respectively, including hypotaurine and 4-aminophenol (m/z 280), histamine and cystathionine (m/z 282), etc. These indicate that the 5-AIQC-tagging method has wide suitability for isomers and analytes having the same ions at unit mass when they have distinctive chromatographic behavior. However, higher resolution mass spectrometers will be required for analytes having the same unit mass and similar chromatographic behavior; some extra chromatographic resolution measures may also be needed for some isomers having similar fragmentation patterns in mass spectrometry.

**Figure 4:**
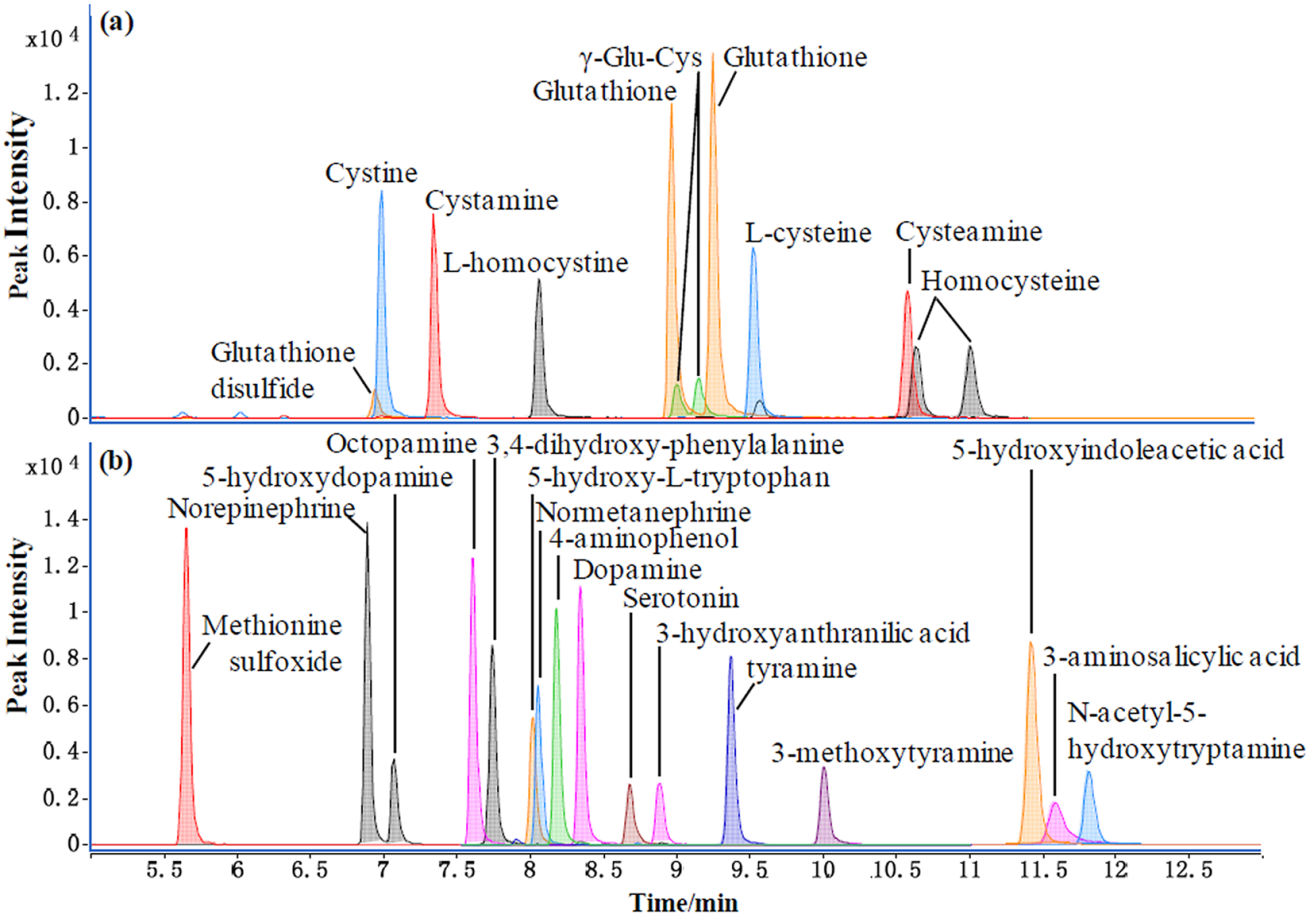
UHPLC-MS/MS chromatograms for the 5-AIQC-tagged oxidation-prone amino analytes including (**a**) these containing thiol and disulfide groups and (**b**) the aromatic metabolites from three aromatic amino acids (phenylalanine, tyrosine and tryptophan).

This method further facilitated simultaneous quantification of 22 sulfur-containing analytes together with some other oxidation-prone aromatic analytes. In particular, the method enabled simultaneous quantification of a number of thiols and disulfides in the same sample in an “one-pot” manner (Table 1, Fig. 4a). To the best of our knowledge, such approach has not been reported previously. It is important to note that aromatic amines such as 3-aminosalicylic acid, 4-aminohippuric acid, 3-aminobenzoic acid and 4-aminobenzoic acid were also readily derivatized and hence quantified by our 5-AIQC approach but not by 6-AQC method^37^ (Table 1). Furthermore, our method enables simultaneous quantification of many oxidation-prone metabolites including dopamine and tyramine metabolites together with these containing thiol and disulfide groups including cysteine-containing metabolites (Table 1, Fig. 4b). The results have also indicated that this 5-AIQC-based method is also applicable for quantification of small peptides including dipeptides (L-carnosine, L-anserine, Ala-Trp, Ala-Leu, Leu-Pro, γ-Glu-Cys) and tripeptides (GSH, GSSG) (Table 1, Supplementary Fig. S6).

**Figure 5:**
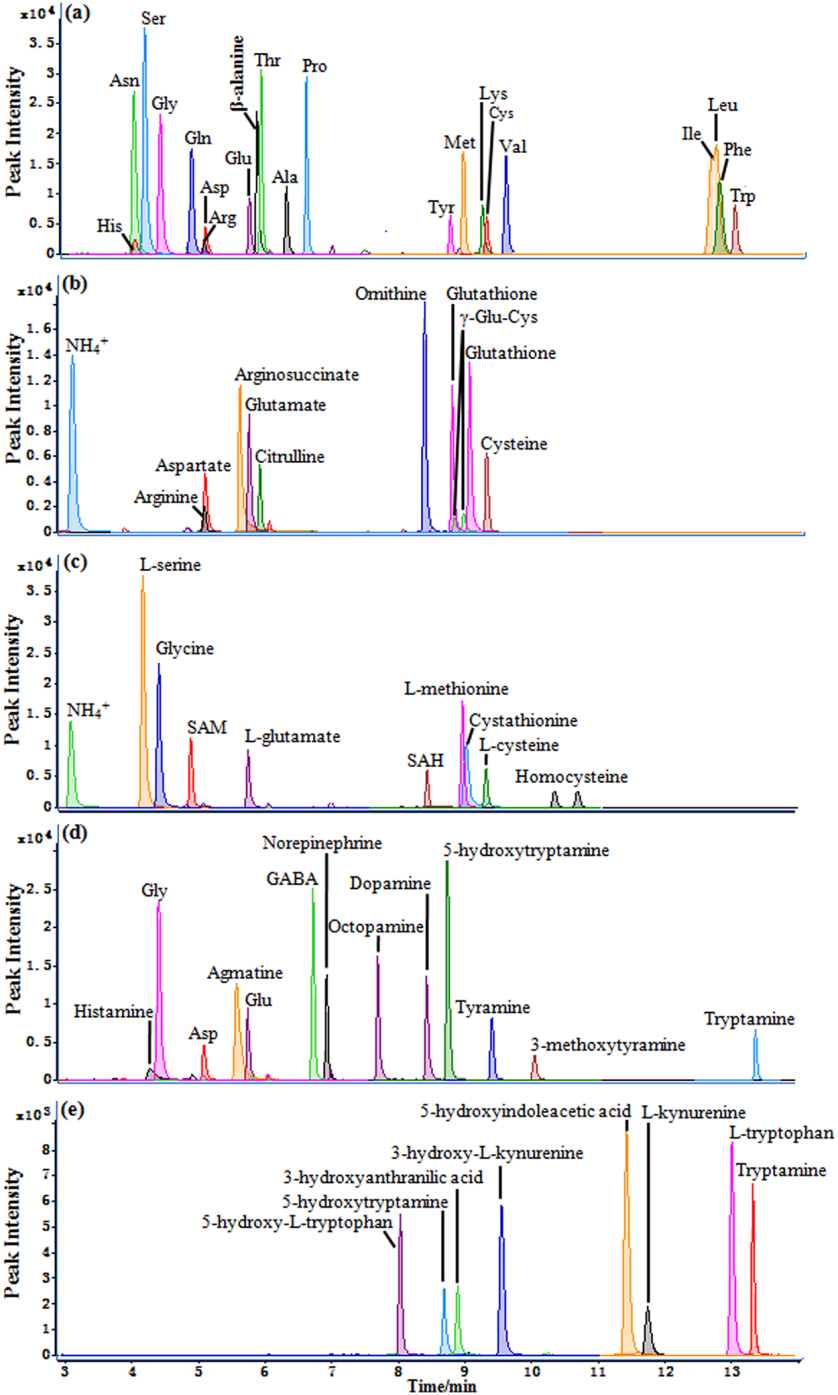
UHPLC-MS/MS chromatograms for the 5-AIQC-tagged amino metabolites in multiple metabolic pathways including (**a**) protein biosynthesis/degradation, (**b**) urea cycle, (**c**) folate-associated homocysteine metabolism, (**d**) biosynthesis of monoamine neurotransmitters and (**e**) tryptophan-mediated kynurenine pathway.

In a single run, moreover, this method enabled simultaneous quantification of multiple metabolites having important functions with the coverage of more than twenty metabolic pathways (Fig. 5, Supplementary Table S2). Quantification of proteinogenic amino acids will be vital for understanding protein biosynthesis/degradation (Fig. 5a) whilst quantification of the arginine-metabolism-related metabolites is important for quantitative understanding the urea cycle (or ornithine cycle) (Fig. 5b). Quantification of cysteine metabolism and the folate-related homocysteine metabolism was also highlighted by multiple intermediates in such metabolic pathway including cysteine, L-methionine, SAM, SAH, homocysteine and cystathionine (Fig. 5c). Furthermore, monoamine neurotransmitters including amino acids themselves, metabolites derived from both aliphatic amino acids (e.g., agmatine) and aromatic ones (e.g., catecholamines) including phenylalanine-tyrosine metabolism and tryptophan-metabolism mediated 5-hydroxytryptamine pathway (Fig. 5d–e). The coverage of the tryptophan-metabolism mediated kynurenine pathway was reflected by eight major metabolites in the pathway (Fig. 5e) whilst such coverage of polyamine pathway was well highlighted by spermine, putrescine, cadaverine and spermidine (Supplementary Table S2).

However, we found that 5-AIQC was not suitable for derivatization of adenine, amide, guanido and urea groups as in the case of 6-AQC^37^.

### Sensitivity, precision, accuracy and recovery for this quantification method

To validate this method, 95 amino compounds with their concentration in the range of 0.02–200 μM were employed to respectively represent proteinogenic and non-proteinogenic amino acids, modified amino acids, small peptides, aliphatic and aromatic amines, oxidation-prone analytes (such as thiols, disulfides and catecholamines). Their mixtures (Mix1-Mix9) were prepared in volumetric flasks from solution of each standard with gradual dilution of the stock solution using phosphate buffer (0.1 M, pH7.0) (Supplementary Table S1) and used for method validation.

The chromatographic reproducibility was evaluated by computing the retention time of each analyte obtained over 3 days using the mixed analytes Mix2, Mix5, Mix6 and Mix7 representing high, intermediate and low concentration situations, respectively (Supplementary Table S1). The intra-day RSDs of the retention times for 95 amino compounds were all below 5% (Supplementary Table S3) and the inter-day RSDs were about 1–6.8%.

Sensitivity was assessed for all 124 metabolites by determination of the limit of detection (LOD) and quantification (LOQ) for amino analytes on column. Linearity of detection response was excellent for all analytes in the concentration range of 0.0002–2 μM (on column) with R^2^ well above 0.99 (Table 1). Amongst 124 analytes tested here, only procaine had LOD above 50 fmol. The LOD was below 32 fmol (on column) for the rest 123 analytes, below 9.5 fmol for 108 analytes, below 5 fmol for 98 analytes and sub-fmol for 26 analytes (Table 1). When the Jet Stream ion source and iFunnel technology was jointly employed (with an Agilent 6495 Mass Spectrometer), sensitivity was further improved (up to 8 folds) with the LOD reached sub-fmol level for most 95 analytes tested (Supplementary Table S4).

Our method had superior LODs for all analytes when compared with the results from the 6-AQC approach^37^ under the same analytical conditions (Table 1). Noticeably, our method was 5 times more sensitive for His, Thr, Asp, taurine and ethanolamine whilst 10 times more sensitive for L-proline, ethylamine, and 4-aminophenol than the 6-AQC method (Table 1). The only exception was homocysteine that showed only slightly lower sensitivity. Such sensitivity enhancement is probably due to the fact that isoquinoline is more basic (p*Ka* ~ 5.40) than quinoline (p*Ka* ~ 4.95)^55^. Consistently, our data measured from an NMR method^56^ showed the p*Ka* values of 5.31 ± 0.07 and 4.95 ± 0.03, respectively, for the 5-aminoisoquinoline and 6-aminoquinoline ring nitrogen (Supplementary Fig. S7).

It is particularly important to note that our 5-AIQC approach can be used to derivatize numerous aromatic amines successfully including 3-aminosalicylic acid, 4-aminohippuric acid, 3-aminobenzoic acid and 4-aminobenzoic acid. In contrast, 6-AQC approach cannot be used to analyze them^37^. Although 4-aminophenol can be analyzed by both 5-AIQC and 6-AQC methods, LOD was more than an order of magnitude (17 times) lower for 5-AIQC method than 6-AQC approach (Table 1). Nonetheless, both 5-AIQC and 6-AQC failed to tag the amino group of adenosine.

The intra-and inter-day variations for quantification of analytes were assessed by using four mixed standard solutions (i.e., Mix2, Mix5, Mix6, and Mix7) representing high, intermediate and low concentration cases respectively. In any case, both the intra- and inter-day RSDs were below 15% (Supplementary Table S3) for most analytes except cystine, sarcosine and 2-aminoisobutyrate. Cystine had inter-day RSDs just over 16% at intermediate to high concentration. However, the intra- and inter-day variations for both sarcosine and 2-aminoisobutyrate were surprisingly poor ranging from 27% to 90% (Supplementary Table S3) though ionization efficiency of them was not problematic and such remained to be understood.

Accuracy for the simultaneous quantification of these amino analytes were evaluated by calculated recoveries from three mixed standard solutions (Mix1, Mix4 and Mix6), respectively, in which Mix4 was spiked. The results showed that such recoveries for most analytes were about 88–116% (Table 2) with most of the oxidation-prone compounds around 88.5–110.9%. Tyr had such recovery over 120% at mediate to high concentrations. We have also found that such recoveries were about 80–120% for 43 and 29 representative analytes in rat urine and serum samples, respectively. The obvious exceptions were again observed for sarcosine and 2-aminoisobutyric acid in rat urine with virtual recoveries of 193.8% and 189.2%, respectively (Table 2) for unknown reasons though this might be related to their poor inter- and intra-day quantification precision.

**Table 2:** Recoveries for 95 representative amino analytes with low (L), intermediate (M) and high concentration (H) from standard mixtures (Stds, n = 5), human renal tumor and adjacent non-involved tissues (ANIT) (n = 6), rat urine and serum (n = 6) samples.

	Stds (L)	Stds (M)	Stds (H)	Renal tumor	Renal ANIT	Rat urine	Rat serum
L-Asparagine	111.8(1.0)	113.2(6.3)	112.9(4.4)	**116.2**(**3.0**)	**112.3**(**0.5**)	**119.7**(**6.8**)	**118.7**(**6.5)**
L-Histidine	115.6(2.8)	107.8(10.6)	99.3(5.5)	**106.6**(**4.4**)	**111.7**(**2.1**)	**109.7**(**7.6**)	**124.9**(**7.7**)
L-Serine	101.0(5.4)	105.5(12.5)	107.5(3.5)	**86.4**(**1.5**)	**80.8**(**0.9**)	**117**(**11.9**)	**120.2**(**6.1**)
Glycine	109.7(5.0)	99.6(4.6)	105.4(3.2)	**86.4**(**1.5**)	**80.8**(**0.9**)	**105.2**(**6.6**)	**96.4**(**8.4**)
L-Glutamine	104.1(2.2)	111.5(8.5)	107.6(6.1)	**114.3**(**1.1**)	**109.4**(**2.5**)	**106.1**(**9.1**)	**98.9**(**10.1**)
L-Arginine	101.3(2.0)	89.2(6.4)	91.6(4.2)	**115.0**(**3.1**)	**117.6**(**2.6**)	**105.6**(**16.1**)	**109.4**(**10.7**)
L-Aspartic acid	98.9(4.6)	107.9(6.1)	107.9(0.6)	**116.0**(**1.5**)	**114.1**(**2.0**)	**117.1**(**8.8**)	**119.8**(**1.7**)
L-Glutamic acid	116.1(2.1)	113.8(5.0)	108.1(0.9)	**114.4**(**3.2**)	**119.9**(**2.2**)	**113.2**(**10.7**)	**108.5**(**6.5**)
L-Threonine	110.3(4.5)	109.3(2.9)	109.3(0.2)	**117.6**(**2.4**)	**116.6**(**1.7**)	**115.5**(**11.7**)	**114.5**(**8.4**)
L-Alanine	102.9(2.2)	107.0(6.3)	111.3(3.1)	**120.8**(**2.4**)	**120.4**(**1.3**)	**112.1**(**7.2**)	**118.6**(**8.1**)
L-Proline	111.1(0.8)	108.2(5.3)	107.4(3.7)	**112.5**(**3.0**)	**115.1**(**2.0**)	**106.5**(**5.9**)	**101.9**(**7.1**)
L-Tyrosine	113.7(4.7)	126.6(6.3)	135.7(2.1)	**131.7**(**2.2**)	**116.1**(**4.2**)	**99.7**(**4.6**)	**119.4**(**6.3**)
L-Methionine	107.9(2.2)	106.5(6.4)	108.7(4.7)	**108.6**(**1.8**)	**105.6**(**2.2**)	**100.8**(**8.4**)	**108.1**(**6.9**)
L-Lysine	106.4(2.8)	106.3(5.1)	106.7(3.5)	**114.3**(**1.9**)	**110.5**(**6.3**)	**109.3**(**9.3**)	**110.5**(**10.0**)
L-Valine	109.2(1.2)	107.4(4.2)	108.8(4.7)	**112.1**(**1.8**)	**110.0**(**1.9**)	**119.3**(**1.9**)	**105.4**(**7.5**)
L-Isoleucine	110.0(2.4)	113.2(7.3)	108.8(3.5)	**114.9**(**2.7**)	**108.1**(**1.3**)	**84.9**(**14.2**)	**119.3**(**4.3**)
L-Leucine	99.6(4.5)	104.2(6.6)	112.5(3.4)	**113.9**(**3.7**)	**107.1**(**3.3**)	**99.3**(**16.4**)	**96.2**(**13**)
DL-Phenylalanine	103.7(2.5)	107.3(7.8)	107.9(3.3)	**108.2**(**2.3**)	**115.7**(**0.7**)	**115.9**(**1.9**)	**105.4**(**6.1**)
L-Tryptophan	103.3(4.5)	101.6(7.5)	103.4(4.8)	**105.8**(**2.8**)	**103.7**(**1.9**)	**117.3**(**1.3**)	**115.6**(**4.3**)
D-Homoserine	110.0(3.9)	112.9(4.9)	111.6(1.3)	*116*.*2*(*1*.*7*)	*109*.*3*(*3*.*4*)	*105*.*6*(*7*.*4*)	*103*.*4*(*5*.*3*)
β-alanine	108.1(2.0)	105.3(4.4)	106.6(0.7)	**114.8**(**4.3**)	**114.7**(**2.7**)	**101.1**(**8.3**)	*110*.*0*(*6*.*7*)
L-Citrulline	113.4(3.8)	109.9(5.6)	108.4(2.3)	**114.0**(**5.4**)	**108.7**(**3.5**)	**117.1**(**3.9**)	**103.9**(**6.7**)
L-Homoarginine	102.4(6.2)	97.0(7.4)	79.9(8.6)	*119*.*5*(*0*.*5*)	*108*.*6*(*8*.*8*)	*103*.*5*(*5*.*0*)	*82*.*7*(*6*.*9*)
γ-aminobutyric acid	112.6(2.4)	105.0(3.9)	108.2(2.5)	**111.4**(**5.0**)	**107.8**(**0.5**)	**112.6**(**3.1**)	*106*.*6*(*5*.*5*)
L-Homocitrulline	110.8(1.4)	112.7(3.4)	108.9(3.3)	*115*.*4*(*5*.*4*)	*115*.*7*(*1*.*9*)	*116*.*5*(*6*.*0*)	*108*.*0*(*5*.*3*)
L-2-aminoadipic acid	112.7(2.4)	111.8(10.8)	111.5(4.1)	*110*.*8*(*4*.*6*)	*114*.*5*(*0*.*9*)	*108*.*7*(*10*.*0*)	*104*.*2*(*4*.*4*)
DL-3-aminoisobutyrate	110.9(3.3)	107.5(4.5)	106.6(1.5)	**110.5**(**3.1**)	**108.2**(**2.8**)	**108.3**(**4.8**)	*96*.*7*(*5*.*9*)
2-Aminoisobutyric acid	47.6(8.8)	163.7(11.9)	157.1(8.4)	*144*.*3*(*1*.*6*)	*113*.*5*(*2*.*4*)	**189.2**(**1.2**)	*158*.*0*(*0*.*4*)
5-Aminovaleric acid	117.4(1.8)	111.3(5.9)	110.6(2.2)	*115*.*3*(*2*.*0*)	*110*.*1*(*3*.*4*)	**114.1**(**3.3**)	*105*.*5*(*3*.*7*)
L-2-Aminobutyric acid	108.8(3.2)	107.5(6.3)	109.6(2.0)	**111.7**(**4.6**)	**108.5**(**2.8**)	**86.8**(**6.7**)	**106.8**(**4.9**)
2,4-diaminobutyric acid	113.1(1.8)	114.8(5.9)	113.8(0.7)	*115*.*8*(*4*.*4*)	*110*.*6*(*2*.*0*)	*100*.*5*(*5*.*0*)	*101*.*9*(*4*.*6*)
DL-2,6-diaminopimelate	104.2(3.1)	108.2(5.7)	114.3(4.9)	*111*.*7*(*0*.*4*)	*101*.*0*(*2*.*4*)	*106*.*4*(*6*.*4*)	*104*.*7*(*4*.*9*)
L-Ornithine	102.2(2.4)	108.2(5.6)	111.4(1.3)	**111.9**(**1.6**)	**108.1**(**4.8**)	**120.2**(**1.1**)	**113.5**(**4.2**)
6-Aminocaproic acid	104.0(2.6)	106.3(5.6)	106.1(2.4)	*107*.*1*(*1*.*2*)	*103*.*3*(*2*.*9*)	*99*.*6*(*3*.*2*)	*118*.*6*(*3*.*7*)
L-Norvaline	107.5(1.3)	106.3(7.0)	108.8(3.7)	*117*.*9*(*2*.*7*)	*112*.*5*(*4*.*1*)	*107*.*2*(*7*.*1*)	*108*.*7*(*4*.*9*)
D–(−)-α-Phenylglycine	99.2(3.5)	107.9(3.7)	107.9(4.5)	*109*.*5*(*3*.*1*)	*104*.*1*(*2*.*9*)	*103*.*2*(*4*.*3*)	*117*.*0*(*5*.*9*)
L-Kynurenine	106.2(4.7)	105.8(1.7)	110.5(2.8)	*111*.*6*(*1*.*9*)	*110*.*3*(*3*.*4*)	*106*.*5*(*13*.*5*)	*115*.*0*(*8*.*2*)
L-Norleucine	105.4(4.8)	102.5(2.8)	104.8(3.6)	*111*.*6*(*1*.*9*)	*109*.*1*(*1*.*9*)	*109*.*2*(*4*.*0*)	*97*.*3*(*4*.*2*)
(−)-Norepinephrine	107.2(2.1)	108.0(7.2)	108.7(2.4)	*111*.*1*(*3*.*1*)	*104*.*2*(*2*.*8*)	*103*.*0*(*8*.*1*)	*102*.*1*(*3*.*4*)
(±)-Octopamine	113.2(6.9)	110.8(5.1)	110.7(1.3)	*112*.*9*(*1*.*0*)	*111*.*6*(*2*.*5*)	*114*.*4*(*9*.*9*)	*110*.*0*(*14*.*1*)
Dopamine	112.3(2.4)	105.9(6.7)	109.7(1.4)	*108*.*0*(*3*.*6*)	*109*.*1*(*4*.*6*)	*116*.*6*(*1*.*2*)	*112*.*3*(*4*.*2*)
Tyramine	106.4(2.7)	101.7(5.1)	106.0(3.0)	*104*.*2*(*3*.*7*)	*107*.*5*(*5*.*2*)	**122.1**(**2.5**)	*93*.*4*(*3*.*1*)
3-Methoxytyramine	105.1(2.1)	103.4(5.8)	107.4(0.5)	*108*.*6*(*4*.*8*)	*107*.*7*(*5*.*2*)	*111*.*4*(*11*.*4*)	*106*.*0*(*5*.*7*)
Tryptamine	111.1(3.9)	109.4(5.0)	108.7(5.2)	*110*.*0*(*2*.*8*)	*118*.*7*(*1*.*6*)	*119*.*7*(*1*.*8*)	*79*.*1*(*0*.*9*)
4-Aminophenol	107.2(1.3)	107.8(6.3)	109.4(3.2)	*106*.*8*(*3*.*0*)	*107*.*0*(*1*.*9*)	*86*.*2*(*3*.*5*)	*116*.*9*(*3*.*0*)
*4*-Aminohippuric acid	117.7(6.2)	111.4(19.6)	100.4(5.3)	*107*.*3*(*0*.*5*)	*109*.*3*(*6*.*3*)	*103*.*2*(*6*.*4*)	*100*.*8*(*8*.*5*)
3-Aminobenzoic acid	114.4(1.9)	99.8(6.3)	101.9(8.2)	*104*.*1*(*2*.*3*)	*99*.*9*(*6*.*9*)	*95*.*1*(*0*.*8*)	*97*.*0*(*0*.*9*)
3-Aminosalicylic acid	113.3(2.1)	106.9(4.6)	106.4(3.8)	*103*.*3*(*3*.*0*)	*115*.*0*(*2*.*6*)	*120*.*8*(*3*.*0*)	*104*.*1*(*10*.*2*)
4-Aminobenzoic acid	104.8(5.6)	105.8(5.3)	108.2(1.5)	*105*.*0*(*2*.*6*)	*110*.*0*(*2*.*3*)	*82*.*4*(*0*.*9*)	*84*.*3*(*1*.*0*)
L-Cysteic acid	100.5(1.0)	105.9(7.4)	113.6(3.2)	*120*.*1*(*1*.*6*)	*110*.*5*(*3*.*5*)	*101*.*0*(*6*.*1*)	*106*.*8*(*7*.*2*)
Taurine	116.4(3.0)	105.5(9.0)	107.5(3.9)	**114.3**(**1.9**)	**127.6**(**3.2**)	**108.6**(**3.1**)	**93.5**(**15.0**)
Hypotaurine	113.5(2.4)	106.0(6.7)	103.0(1.6)	**109.3**(**2.5**)	**106.8**(**2.7**)	**120.6**(**2.2**)	*105*.*5*(*2*.*6*)
DL-Methionine sulfone	109.4(0.5)	112.7(4.5)	111.2(2.2)	*114*.*0*(*3*.*7*)	*109*.*9*(*2*.*1*)	*103*.*2*(*6*.*4*)	*103*.*8*(*3*.*9*)
DL-methionine sulfoxide	118.9(1.7)	112.4(3.7)	112.5(5.4)	*116*.*7*(*4*.*6*)	*111*.*3*(*0*.*7*)	*108*.*4*(*5*.*9*)	*107*.*5*(*5*.*0*)
Glutathione disulfide	88.5(4.3)	95.9(15.5)	97.7(2.9)	**94.3**(**6.6**)	**99.0**(**8.2**)	*104*.*0*(*5*.*8*)	*95*.*4*(*3*.*6*)
L-Cystine	103.2(3.4)	101.3(5.4)	107.1(2.8)	**108.5**(**2.9**)	**117.3**(**1.7**)	**84.6**(**2.2**)	**90.7**(**1.4**)
Cystamine	106.2(0.7)	102.5(9.5)	104.0(4.0)	*105*.*7*(*1*.*8*)	*105*.*3*(*3*.*3*)	**89.1**(**0.7**)	*84*.*1*(*2*.*6*)
L-Homocystine	101.0(6.7)	102.5(6.8)	105.5(4.0)	*108*.*3*(*4*.*7*)	*104*.*3*(*3*.*2*)	*100*.*2*(*2*.*3*)	*83*.*5*(*1*.*6*)
Cystathionine	104.7(2.0)	112.0(8.0)	109.3(4.8)	*118*.*3*(*5*.*4*)	*83*.*2*(*10*.*7*)	*108*.*3*(*3*.*8*)	*87*.*7*(*3*.*4*)
S-(2-Aminoethyl)-L-Cys	104.3(1.9)	110.0(7.4)	109.0(2.5)	*110*.*4*(*2*.*5*)	*104*.*6*(*3*.*0*)	*112*.*4*(*1*.*0*)	*105*.*9*(*4*.*7*)
L-Cysteine	102.9(1.7)	110.9(7.5)	107.8(2.4)	**108.1**(**1.2**)	**102.9**(**2.1**)	**109.9**(**3.8**)	**99.9**(**3.5**)
Djenkolic Acid	94.9(6.3)	103.7(5.7)	105.3(7.8)	*102*.*7*(*7*.*8*)	*97*.*0*(*5*.*4*)	*111*.*8*(*2*.*2*)	*109*.*6*(*3*.*5*)
Cysteamine	108.7(1.9)	104.4(4.7)	105.8(1.6)	*111*.*3*(*2*.*2*)	*108*.*7*(*4*.*1*)	*92*.*1*(*0*.*7*)	*90*.*4*(*3*.*6*)
DL-Ethionine	104.9(0.8)	109.2(6.5)	108.1(3.5)	*108*.*5*(*1*.*2*)	*108*.*6*(*2*.*6*)	*106*.*1*(*6*.*6*)	*108*.*2*(*3*.*3*)
DL-Homocysteine	109.0(2.6)	110.3(6.3)	108.3(3.8)	*108*.*3*(*4*.*1*)	*107*.*6*(*6*.*9*)	*100*.*8*(*5*.*9*)	*92*.*8*(*7*.*0*)
Glutathione	93.2(12.1)	104.9(8.8)	105.1(10.9)	*105*.*2*(*13*.*6*)	*112*.*8*(*10*.*6*)	*95*.*0*(*9*.*9*)	*82*.*8*(*7*.*2*)
L-Carnosine	104.9(6.7)	101.4(10.3)	97.7(7.6)	*134*.*3*(*5*.*8*)	*138*.*0*(*6*.*3*)	*103*.*0*(*8*.*0*)	*95*.*6*(*6*.*3*)
Ala-leu	113.5(9.3)	108.0(4.2)	109.0(2.9)	*107*.*2*(*2*.*6*)	*105*.*1*(*2*.*0*)	*113*.*7*(*5*.*2*)	*104*.*3*(*4*.*6*)
Ala-Trp	105.3(4.3)	109.6(6.5)	108.6(4.7)	*116*.*7*(*0*.*9*)	*112*.*4*(*1*.*9*)	*113*.*6*(*3*.*1*)	*116*.*6*(*1*.*9*)
*trans*-4-Hydroxy-L-Pro	106.2(7.3)	107.9(8.1)	116.6(3.9)	**98.8**(**2.5**)	**100.2**(**3.1**)	*84*.*3*(*6*.*6*)	*95*.*6*(*13*.*1*)
*O*-PE	114.4(9.0)	107.3(9.4)	106.9(5.6)	*110*.*3*(*1*.*7*)	*114*.*9*(*1*.*1*)	*108*.*0*(*2*.*6*)	*99*.*5*(*1*.*8*)
Sarcosine	18.4(7.3)	76.8(16.1)	75.2(11.5)	*110*.*1*(*7*.*4*)	*86*.*4*(*5*.*1*)	**193.8**(**2.7**)	*154*.*4*(*4*.*4*)
3-Methyl-L-histidine	107.2(4.9)	96.4(2.9)	91.1(6.3)	*112*.*8*(*1*.*3*)	*104*.*5*(*11*.*7*)	*106*.*3*(*9*.*9*)	**107**(**9.3**)
1-Methyl-L-histidine	90.9(12.1)	90.9(14.5)	88.5(2.1)	*106*.*9*(*9*.*7*)	*106*.*9*(*3*.*6*)	*109*.*6*(*12*.*2*)	**120.3**(**2.5**)
ADMA	114.2(16.8)	112.8(10.2)	108.2(4.7)	*102*.*1*(*9*.*6*)	*104*.*8*(*16*.*6*)	*108*.*1*(*4*.*1*)	*87*.*7*(*4*.*3*)
*O*-acetyl-L-serine	106.3(3.7)	107.8(5.0)	107.7(2.9)	*110*.*2*(*2*.*7*)	*105*.*5*(*2*.*6*)	*103*.*4*(*3*.*8*)	*120*.*3*(*4*.*3*)
Nα-Acetyl-L-lysine	109.5(0.9)	109.9(7.1)	112.6(2.8)	*111*.*9*(*3*.*3*)	*106*.*5*(*2*.*4*)	**114.4**(**3.8**)	*100*.*3*(*5*.*3*)
DL-5-Hydroxylysine	103.1(3.0)	105.0(8.5)	108.0(3.0)	**111.2**(**2.8**)	**103.0**(**2.7**)	*105*.*9*(*5*.*7*)	*103*.*9*(*3*.*6*)
5-Hydroxy-L-tryptophan	106.3(4.9)	103.5(1.6)	103.4(3.0)	*104*.*0*(*3*.*8*)	*104*.*9*(*2*.*1*)	*109*.*2*(*2*.*3*)	*103*.*6*(*3*.*0*)
4-Hydroxy-L-isoleucine	88.6(2.1)	117.2(2.7)	124.0(1.4)	*117*.*9*(*8*.*0*)	*97*.*4*(*1*.*6*)	*110*.*6*(*4*.*6*)	*120*.*6*(*3*.*5*)
Ethanolamine	110.2(2.4)	104.0(8.7)	103.5(3.3)	**113.0**(**2.4**)	**116.4**(**2.9**)	**114.6**(**2.2**)	**102.6**(**5.8**)
Methylamine	115.3(2.2)	107.4(6.8)	110.9(5.8)	*114*.*2*(*1*.*2*)	*113*.*1*(*3*.*2*)	**110.2**(**15.2**)	**110.3**(**6.6**)
Agmatine	108.4(7.2)	105.6(4.5)	113.5(1.6)	*118*.*4*(*0*.*3*)	*113*.*8*(*3*.*0*)	*110*.*8*(*6*.*2*)	*100*.*2*(*2*.*6*)
Ethylamine	116.0(0.8)	107.5(5.8)	108.2(3.7)	*108*.*5*(*1*.*4*)	*111*.*6*(*1*.*3*)	**107.5**(**1.4**)	*105*.*7*(*6*.*4*)
Putrescine	105.2(3.3)	104.5(6.2)	105.1(7.2)	*109*.*4*(*1*.*5*)	*110*.*9*(*6*.*4*)	**110.1**(**2.2**)	*104*.*2*(*3*.*0*)
Cadaverine	103.3(3.8)	105.9(3.0)	106.5(1.4)	*106*.*8*(*1*.*6*)	*110*.*1*(*2*.*2*)	**105.7**(**2.0**)	*106*.*7*(*5*.*1*)
Spermine	99.2(3.2)	105.9(9.9)	101.1(9.8)	*100*.*0*(*8*.*9*)	*103*.*1*(*3*.*6*)	**82.3**(**0.5**)	*102*.*7*(*7*.*0*)
Prolinamide	110.3(7.2)	104.8(5.6)	109.4(4.6)	*109*.*2*(*2*.*5*)	*104*.*7*(*4*.*6*)	*110*.*2*(*5*.*1*)	*98*.*6*(*5*.*5*)
Allantoin	98.1(0.8)	98.9(6.5)	113.7(9.4)	*113*.*6*(*10*.*5*)	*108*.*7*(*12*.*3*)	**120.1**(**1.3**)	*100*.*0*(*11*.*9*)
5-Hydroxydopamine	116.1(6.2)	108.9(4.4)	102.9(7.5)	*105*.*5*(*3*.*7*)	*106*.*1*(*3*.*9*)	*94*.*4*(*4*.*5*)	*103*.*1*(*6*.*0*)
3,4-dihydroxy-DL-Phe	116.2(0.4)	106.7(4.9)	106.8(3.8)	*108*.*7*(*5*.*4*)	*108*.*4*(*2*.*4*)	*105*.*3*(*10*.*7*)	*110*.*4*(*2*.*7*)
DL-Normetanephrine	112.3(6.0)	108.2(6.5)	107.9(3.3)	*107*.*1*(*1*.*8*)	*103*.*5*(*3*.*6*)	*110*.*5*(*1*.*3*)	*107*.*0*(*4*.*3*)
1,3-Diaminopropane	109.8(2.1)	107.8(5.8)	111.6(6.6)	*111*.*6*(*1*.*0*)	*109*.*5*(*2*.*8*)	**106.1**(**0.9**)	*103*.*0*(*3*.*6*)
1,2-Diaminopropane	104.8(1.5)	105.2(7.0)	106.2(1.2)	*106*.*2*(*4*.*2*)	*102*.*3*(*3*.*4*)	*100*.*6*(*1*.*3*)	*90*.*3*(*3*.*6*)
L-Tryptophanamide	104.0(3.1)	105.4(9.0)	107.8(3.8)	*111*.*2*(*3*.*1*)	*111*.*1*(*1*.*6*)	*100*.*3*(*2*.*6*)	*112*.*0*(*3*.*9*)

Data in parathesis are RSD (%); data in bold letters were from metabolites detected in real sample whereas these in italics were not. ADMA: Asymmetric dimethylarginine; Cys: cysteine, Pro: proline; *O*-PE: *O*-Phosphorylethanolamine; Phe: phenylalanine.

### Quantification of amino-group containing metabolites in haemolymph of silkworm (*Bombyx mori* L.)

We further applied this newly developed method to analyze the amino metabolites in the silkworm haemolymph at three developmental stages (Table 3). 45 amino metabolites were quantified including 20 proteinogenic and 11 non-proteinogenic amino acids (4-hydroxy-proline, 1-methyl-histidine, 3-methyl-histidine, ornithine, citrulline, β-alanine, γ-aminobutyric acid, 2-aminobutyric acid, 3-aminoisobutyric acid, 2-aminoadipic acid, Nε,Nε,Nε-trimethyllysine), 6 sulfur-containing metabolites (methionine sulfoxide, methionine sulfone, cysteine, cystine, GSSG, cystathionine), 2 polyamines (putrescine, 1,3-diaminopropane), 2 catecholamines (3,4-dihydroxy-phenylalanine, dopamine) and 2 ethanolamines (ethanolamine and o-phosphorylethanolamine) (Table 3). Amongst them, many amino metabolites were not reported in the classical studies of silkworm haemolymph with ion-exchange and paper chromatographic^57^ and/or more recent NMR studies^7, 58^ including 3-methyl-histidine, 2-aminobutyric acid, 3-aminoisobutyric acid, 2-aminoadipic acid, methionine sulfone, GSSG, 1,3-diaminopropane and 3,4-dihydroxy-phenylalanine (Table 3). This is probably due to much higher sensitivity of our present method than these used previously. The rich amino metabolites in silkworm haemolymph clearly showed concentration variations with the developmental processes (Table 3) as reported previously^7^ reflecting the functions of the metabolites for silkworm’s growth, activities and ecdysis in energy metabolism, biosynthesis of proteins^57, 59^ and pigments. These will be discussed in details elsewhere.

**Table 3:** Concentration of amino metabolites (mM) in haemolymph of silkworm (*Bombyx mori* L strain P50) (the first number denotes day and second one instars, e.g, 5d5I: day 5 in the fifth instar; pP: pre-pupa).

Amino metabolites	3d3I	5d5I	pP
1-Deoxynojirimycin	6.136 ± 0.748	5.027 ± 0.608	0.467 ± 0.115
L-Glutamine	10.248 ± 3.674	10.073 ± 1.397	15.326 ± 0.678
L-Asparagine	2.157 ± 0.545	3.179 ± 0.388	2.873 ± 1.058
L-Glutamic acid	0.487 ± 0.179	0.107 ± 0.046	5.166 ± 3.227
L-Aspartic acid	0.071 ± 0.038	0.049 ± 0.013	0.643 ± 0.421
Glycine	6.078 ± 1.382	5.620 ± 0.546	12.242 ± 1.484
L-Alanine	3.065 ± 0.643	3.197 ± 1.194	5.441 ± 1.688
L-Serine	7.318 ± 1.556	10.807 ± 0.892	7.629 ± 0.284
L-Threonine	4.748 ± 1.570	2.998 ± 0.583	7.158 ± 0.777
L-Valine	5.508 ± 1.674	1.570 ± 0.582	7.570 ± 0.401
L-Isoleucine	4.548 ± 0.743	0.880 ± 0.319	8.857 ± 1.393
L-Leucine	3.794 ± 0.602	0.664 ± 0.258	7.287 ± 0.815
L-Proline	2.293 ± 0.866	1.085 ± 0.298	8.104 ± 1.160
L-Methionine	1.067 ± 0.285	0.520 ± 0.140	1.766 ± 0.390
L-Histidine	5.010 ± 1.642	18.247 ± 1.514	38.693 ± 2.239
L-Lysine	11.175 ± 4.222	3.955 ± 0.713	8.848 ± 1.069
L-Arginine	3.514 ± 1.394	0.723 ± 0.145	2.696 ± 0.215
L-Phenylalanine	0.450 ± 0.101	0.465 ± 0.073	1.588 ± 0.240
L-Tyrosine	3.696 ± 0.588	0.110 ± 0.058	4.343 ± 0.944
L-Tryptophan	0.315 ± 0.076	0.098 ± 0.025	1.412 ± 0.323
β-alanine	0.459 ± 0.200	0.256 ± 0.042	0.284 ± 0.046
L-Ornithine	3.660 ± 1.900	9.224 ± 2.090	0.613 ± 0.296
L-Citrulline	0.235 ± 0.038	0.083 ± 0.030	0.013 ± 0.001
4-Hydroxy-L-proline	0.057 ± 0.014	0.146 ± 0.017	0.033 ± 0.004
L-2-aminoadipic acid	0.004 ± 0.001	0.051 ± 0.022	0.041 ± 0.012
γ-Aminobutyric acid	0.003 ± 0.001	0.019 ± 0.006	0.012 ± 0.003
DL-3-aminoisobutyric acid	0.023 ± 0.017	0.057 ± 0.007	0.018 ± 0.003
L-2-Aminobutyric acid	0.047 ± 0.009	0.064 ± 0.014	0.131 ± 0.021
3-hydroxykynurenine	1.662 ± 0.103	0.074 ± 0.005	0.402 ± 0.090
1-Methyl-L-histidine	0.050 ± 0.012	0.014 ± 0.002	0.340 ± 0.054
3-Methyl-L-histidine	0.064 ± 0.022	0.017 ± 0.003	0.022 ± 0.003
Nε,Nε,Nε-trimethyllysine	0.880 ± 0.166	1.270 ± 0.158	1.980 ± 0.197
Dopamine	0.032 ± 0.011	0.006 ± 0.001	0.059 ± 0.018
3,4-dihydroxy-DL-phenylalanine	0.611 ± 0.182	0.847 ± 0.206	1.422 ± 0.328
Cysteine	0.010 ± 0.001	0.020 ± 0.004	0.049 ± 0.013
Cystine	0.098 ± 0.028	0.091 ± 0.027	0.113 ± 0.031
DL-Methionine sulfoxide	0.053 ± 0.022	0.017 ± 0.004	10.881 ± 1.776
DL-Methionine sulfone	0.003 ± 0.000	0.008 ± 0.001	0.019 ± 0.001
Cystathionine	2.632 ± 0.373	2.380 ± 0.472	4.502 ± 0.767
Glutathione disulfide	0.134 ± 0.018	0.028 ± 0.008	0.155 ± 0.032
Putrescine	2.262 ± 0.374	4.366 ± 0.691	15.754 ± 1.940
1,3-Diaminopropane	0.011 ± 0.003	0.021 ± 0.003	0.050 ± 0.005
O-Phosphorylethanolamine	0.209 ± 0.081	0.165 ± 0.031	4.778 ± 0.845
Ethanolamine	0.036 ± 0.006	0.011 ± 0.001	0.043 ± 0.004
NH_4_^+^	0.188 ± 0.118	0.031 ± 0.013	1.151 ± 0.059

## Conclusions

We developed a new and parameter-optimized UHPLC-MS/MS method for simultaneous quantification of the amino-group containing metabolites based on derivatization-assisted sensitivity enhancement by 5-aminoisoquinolyl-N-hydroxysuccinimidyl carbamate (5-AIQC). By using an *N*-ethylmaleimide-based click reaction followed with addition of antioxidants (TCEP and ascorbic acid), our method enabled simultaneous quantification of thiols, disulfides and oxidation-prone metabolites concurrently with other amino analytes in an one-pot manner (and in a single run). This method is also applicable to quantify aromatic amines which cannot be done with the 6-AQC-based method^37^. This 5-AIQC-based method had higher sensitivity than the 6-AQC-based one^37^ for an extensive coverage of analytes including 4 amino saccharides, 20 proteinogenic amino acids, 57 non-proteinogenic amino acids, 17 modified amino acids, 26 aliphatic and 8 aromatic amines, 22 sulfur-containing analytes, 14 monoamine neurotransmitters and 8 small peptides. Amongst them, many sets of isomeric analytes (or having the same ion) were also separable on a common reversed-phase column and quantifiable with tandem-mass spectrometry. This method enables simultaneous quantification of 124 important functional metabolites in more than twenty metabolic pathways such as protein biosynthesis/degradation, gut microbiota metabolism, biosynthesis of arginine, glutathione and catecholamine neurotransmitters, urea cycle, uridine catabolism, polyamine pathway together with the metabolisms of phenylalanine, histidine, tryptophan, cysteine-methionine, taurine-hypotaurine and homocysteine. Our method had excellent precision, accuracy, linearity and recovery for most of analytes including thiols, disulfides and other oxidation-prone metabolites in mixed standards, renal tumor extracts, rat urine and plasma samples. We further applied this method to measure the amino metabolites in hemolymph of silkworms at multiple developmental stages and discovered dozens of metabolites which were not reported previously confirming applicability of this method in cohort studies of biological samples. However, sarcosine and 2-aminoisobutyric acid had unexpected poor behavior in terms of their quantification precision and accuracy with unknown reasons at this stage. As the 6-AQC-based method^37^, this method is not suitable for adenine, guanido and amide groups. Nevertheless, with a unique single-charged fragment ion at m/z 171.0550, it is expected that all the 5-AIQC-derivatized amino compounds can be comprehensively analyzed in a semi-targeted and discovery fashion using UHPLC-QTOF-MS approaches. This will be particularly useful for screening large cohort samples. With many classes of metabolites simultaneously quantified in an one-pot manner, this method may also have useful potentials in clinical chemistry settings as well.

## Methods

### Reagents

HPLC grade methanol and acetonitrile were purchased from TEDIA (Shanghai, China) and Sigma-Aldrich (Shanghai, China), respectively. Na_2_HPO_4_·12H_2_O and NaH_2_PO_4_·2H_2_O, boric acid, sodium hydroxide, ethylenediaminetetraacetic acid (EDTA), dimethylsulfoxide (DMSO) were purchased from Sinopharm Chemical Reagent Co. Ltd (Shanghai, China) all as analytical grade reagents. Formic acid, ascorbic acid, tris(2-carboxyethyl)phosphinehydrochloride (TCEP), N,N’-disuccinimidyl carbonate (DSC), *N*-ethylmaleimide (NEM), 4-*tert*-butylbenzenethiol (tBBT), 5-aminoisoquinoline (5-AIQ) and 6-aminoquinoline (6-AQ) were purchased from Sigma-Aldrich (Shanghai, China) together with 126 analyte standards used here. (see details in Table 1).

### Buffer solutions

Phosphate buffer and borate buffer were prepared in a normal manner with their pH adjusted to 7.0 and 8.8, respectively, using sodium hydroxide solution. Phosphate buffer (0.1 M) contained 10 mM ascorbic acid and 10 mM EDTA whereas borate buffer (0.2 M) contained 20 mM TCEP and 1 mM ascorbic acid.

### Standard Solutions

Each amino-containing analyte standard was weighed accurately and dissolved in aqueous solution of formic acid (0.1%) or phosphate buffer as appropriate. The combined solution from known quantity of these standards gave a stock solution of mixed standards. A series of solutions for mixed standards (Supplementary Table S1) was prepared in volumetric flasks by gradual dilution of the stock solution using phosphate buffer (0.1 M, pH7.0) to make such solutions contain 0.1 M phosphate, 10 mM ascorbic acid and 10 mM EDTA. Solutions of analytes containing thiol and disulfide groups (about 50–500 μM) were prepared in air-tight containers in phosphate buffer (0.1 M, pH7.0) containing 10 mM ascorbic acid and 10 mM EDTA followed with storage at −20 °C until further use.

### Collection and treatments of rat urine and serum samples

The animal experiment was approved by the local committee in the Chinese Academy of Sciences and conducted in accordance with the national guidelines for animal research (Ministry of Science and Technology of China, 2006). Urine and serum samples were from 8-weeks old Wistar rats allowing free access to normal chow and water in a standard manner followed with storage at −80 °C. In 100 μL biological fluids (urine and serum), 300 μL methanol was respectively added directly followed with vortex mixing and 10 min centrifugation (11060 × *g*, 4 °C). The supernatant of each sample was then snap-frozen and stored at −80 °C until further analysis.

### Extracts of human renal cancer tissue samples

The human renal cancer and adjacent non involved tissues from tissue bank at Fudan University Shanghai Cancer Center were used with approval by the local ethic committee (050432-4-1212B). Each tissue sample (about 50 mg) was extracted with 600 μL pre-cooled methanol-water mixture (2:1, v/v) using a tissuelyzer (QIAGEN TissueLyser II, Germany) at 20 Hz for 90 s as previously described^60^. Such extracts were respectively redissolved in 600 μL phosphate buffer (0.1 M, pH7.4) as stock solution for UHPLC-MS/MS analysis.

### Hemolymph Sample Collection and Preparation

Hemolymph samples were obtained from silkworms (*Bombyx mori* L. strain p50) at three developmental stages in a previously reported study^7^. These samples were collected on the day 3 in the third instar (3d3I), day 1 in the fourth instar (1d4I), day 4 in the fourth instar (4d4I), and day 1, 3, 5, 7, 8 in the fifth instar (1d5I, 3d5I, 5d5I, 7d5I, 8d5I) as well as at the pre-pupa stage (pP). All these samples were collected in tubes containing thiourea (as an antioxidant) and stored at −80 °C until analysis. Ten biological replicates were employed in this study. For amino metabolites analysis, each hemolymph sample was individually centrifuged (4000 × g, 4 °C) for 10 minutes to obtain supernatants; 10 μL supernatant from each sample was mixed with 40 *μ*L phosphate buffer (0.1 M, pH7.0), snap-frozen and stored at −80 °C till analysis.

### Synthesis of 5-aminoisoquinolyl-N-hydroxysuccinimidylcarbamate (5-AIQC)

5-Aminoisoquinolyl-N-hydroxysuccinimidylcarbamate (5-AIQC) for tagging amino groups was synthesized (Fig. 1) by drop-wise addition of 5-aminoisoquinoline (5-AIQ) solution (2 mmol in 50 mL ACN) to N,N′-disuccinimidylcarbonate (DSC) solution (3 mmol in 40 mL ACN). This was done over about 2 hours at ambient temperature with magnetic stirring. After further stirring for 24 h and removal of acetonitrile by rotary evaporation, 5-AIQC was obtained as crystals from the concentrated solution through filtration (650 mg, 82% yield). Its ^1^H-NMR and ESI-QTOFMS spectral data are shown in Supplementary Fig. S8.

### Derivatization of amino metabolites by tagging amino group with 5-AIQC

The amino analytes were derivatized individually and in the forms of their mixtures with 5-AIQC in dry acetonitrile (Fig. 2, Supplementary Fig. S1). First, each aliquot of standards (10 μL) or biological samples was vortex-mixed with 80 µL of NEM solution (2.5 mM) in phosphate buffer (0.1 M, pH7.0) containing 10 mM ascorbic acid, 10 mM EDTA and 7% DMSO for 1 min. 10 μL tBBT solution (1 M in DMSO) was added followed with addition of 700 μL borate buffer (0.2 M, pH 8.8) containing 20 mM TCEP and 1 mM ascorbic acid. After vortex-mixing and standing for 2 min, 200 µL 5-AIQC solution was then added and incubated at 55 °C for 10 min. The mixture was cooled down to the ambient temperature and added with 10 µL formic acid followed with storage in air-tight tubes at −20 °C until UHPLC-MS/MS analysis.

### UHPLC-ESI-MS/MS Analysis

UHPLC-MS/MS analyses were conducted on an Agilent UHPLC-MS/MS system consisting of an 1290 UHPLC-system coupled with an Agilent 6460 and 6495 triple-quadrupole mass spectrometer (Agilent Technologies, USA) with Jet Stream ion source in both. The latter also employed iFunnel technology to improve detection sensitivity. MassHunter Workstation software was used for data analysis.

The 5-AIQC-tagged samples (1 μL) were individually injected on an UHPLC column (Agilent Zorbax Eclipse XDB-C18 Rapid Resolution HD, 2.1 × 100 mm, 1.8 μm) with its temperature set to 50 °C. Ultrapure water (MilliQ) and methanol containing 0.1% (v/v) formic acid were used as two mobile phases A and B, respectively, with flow rate of 0.6 mL/min. An optimized gradient elution scheme was employed as 1% B (0–2 min), 1–3.8% B (2–4 min), 3.8–22% B (4–8 min), 22–25% B (8–12 min), 25–60% B (12–13 min), 60–80% B (13–13.51 min) and 80–95% B (13.51–16 min).

Mass spectrometers were operated in the positive ion mode. The MS parameters including source, collision energies and fragmentor voltages were optimized for each analyte by directly infusing the derivatized standard. Gas flow was 10 L/min with gas temperature of 315 °C; nebulizer pressure was 50 psi with temperature of 350 °C and sheath gas flow was 10 L/min. Nozzle voltage was 500 V and capillary voltage was 4000 V. Spectra were acquired in the MRM mode with a common fragment ion at m/z 171 for all analytes. All parameters especially collision energies and fragmentor voltages were optimized for each individual analyte by directly infusing the derivatized individual standard.

### Validation of the UHPLC-ESI-MS/MS Analytical Method for Amino Metabolites

Nine mixed solutions of 95 known standards (amino compounds) were employed for method validation and denoted as Mix1-Mix9 (Supplementary Table S1). Precision for retention times was evaluated by using the retention time of each amino compound in the mixed standards recorded on three different days whilst MassHunter Workstation software (Agilent, USA) was used to calculate the linearity (correlation coefficients), limit of detection (with S/N = 3) and limit of quantification (with S/N = 10). For intra-day and inter-day precision of quantification, four different mixed solutions of known standards (Mix2, Mix5, Mix6 and Mix7) were employed to represent high, intermediate and low concentration situations, respectively (Supplementary Table S1) with each mixed-solution repeatedly analyzed five times per day for three days. Quantification accuracy of the method was measured from the Mix1, Mix4 and Mix6 (representing high, intermediate and low concentration situations, respectively) spiked with an equal volume of Mix4. Recovery of each amino metabolite was measured by using the extracts of renal tumor and adjacent non-involved tissues, and deproteinated rat urine and serum samples spiked with Mix3.

## Electronic supplementary material

Supporting information
