# Supplementary material for: Simultaneous Quantification of Amino Metabolites in Multiple Metabolic Pathways Using Ultra-High Performance Liquid Chromatography with Tandem-mass Spectrometry
Source: Sci Rep. 2017 May 3;7:1423. doi: 10.1038/s41598-017-01435-7 (PMC5431165; doi:10.1038/s41598-017-01435-7)
Supplement: Supplementary file 1 — Supporting information [file 41598_2017_1435_MOESM1_ESM.pdf]

SUPPORTING INFORMATION

Simultaneous Quantification of Amino Metabolites in Multiple Metabolic Pathways Using Ultra-High Performance Liquid Chromatography with Tandem-mass Spectrometry

Jin Wang[†‡||], Lihong Zhou[†||], Hehua Lei[||], Fuhua Hao[||], Xin Liu[†], Yulan Wang[||§], and Huiru Tang[‡*]

[†]*School of Life Science and Technology, Huazhong University of Science and Technology, Wuhan, 430074, China* [‡]*State Key Laboratory of Genetic Engineering, Zhongshan Hospital and School of Life Sciences, Fudan University, Shanghai International Centre for Molecular Phenomics, Collaborative Innovation Center for Genetics and Development, Shanghai 200438, China.* [||]*CAS Key Laboratory of Magnetic Resonance in Biological Systems, State Key Laboratory of Magnetic Resonance and Atomic and Molecular Physics, National Centre for Magnetic Resonance in Wuhan, Wuhan Institute of Physics and Mathematics, Chinese Academy of Sciences, Wuhan, 430071, China*

[§]*Collaborative Innovation Center for Diagnosis and Treatment of Infectious Diseases, Zhejiang University, Hangzhou, 310058, China*

Supplimentary data are available. **Figure S1.** The flowchart for 5-AIQC derivatization of amino analytes with thiol and disulfide groups in one pot. **Figure S2**. Thiol-NEM adducts showed excellent stability under acidic condition by showing no extra hydrolysis within 48h. **Figure S3**. Peak intensity for some 5-AIQC-tagged amino analytes as a function of 5-AIQC-to-analyte molar ratios. **Figure S4.** UHPLC-MS chromatogram for the 5-AIQC adducts of amino analytes showing twin peaks. (A): DL-2,6-diaminopimelic acid; (B) DL-lanthionine; (C): L-glutathione (reduced form); (D): γ-Glu-Cys and (E): DL-homocysteine. **Figure S5** UHPLC-MS/MS chromatograms for 5-AQC-tagged 124 amino analytes. **Figure S6.** UHPLC-MS/MS chromatograms for (A) some small peptides and (B) phenylalanine metabolism and catecholamine biosynthesis. **Figure S7.** pKa measurements for 5-aminoisoquinoline and 6-aminoquinoline (1 mM water solution for both) with ^{1}H NMR method at 298 K. **Figure S8**. Spectroscopic data for 5-AIQC. (a) ^{1}H NMR spectrum in acetonitrile-d_3; (b) QTOF-MS spectrum in positive ion mode (in acetonitrile). **Table S1**. Solutions of mixed standards for method validation. **Table S2**. Metabolic pathways covered by this quantification method. **Table S3.** Intra- and inter-day variations (RSD %) for retention time (RT) and concentration (C) of analytes (n=5). RT_L, RT_M , RT_H, C_L, C_M, C_H were parameters at low (L), intermediate (M) and high (H) concentration range. **Table S4,** Sensitivity enhancement by jointly using the Stream Jet ion sources and iFunnel technology (using an Agilent 6495 triple quad mass spectrometer).

Supplementary Figure S1. The flowchart for 5-AIQC derivatization of amino analytes with thiol and disulfide groups in one pot.

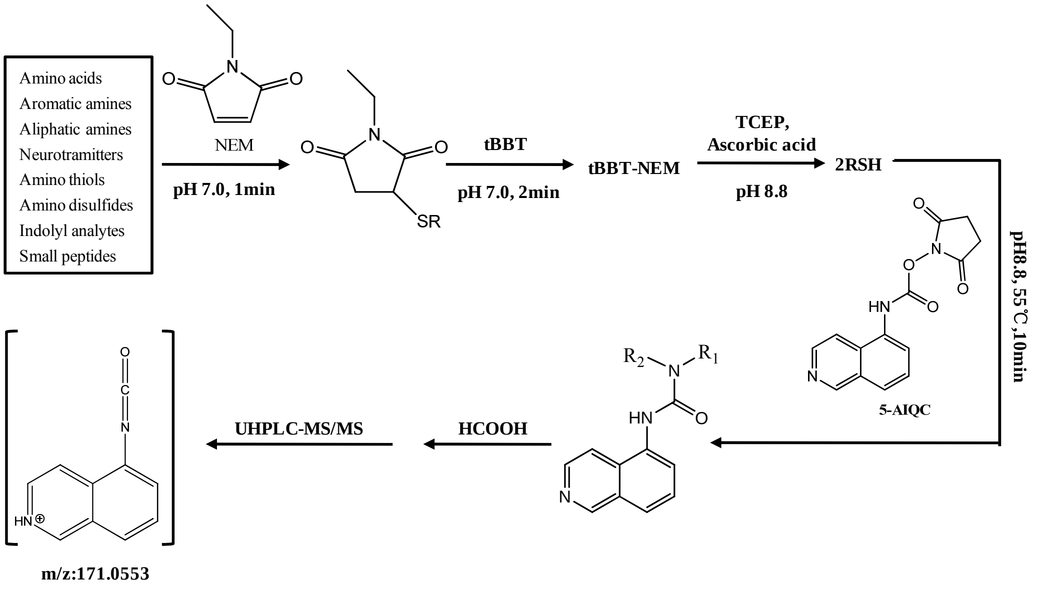

Supplementary Figure S2. Thiol-NEM adducts showed excellent stability under acidic condition by showing no extra hydrolysis within 48h. A_1: peak area of 5-AIQC-RSH-NEM, A_2: peak area of the corresponding hydrolyzed products, 5-AIQC-RSH-NEM-COOH (with ring opening in NEM). Cys: cysteine adduct; GSH: glutathione adduct.

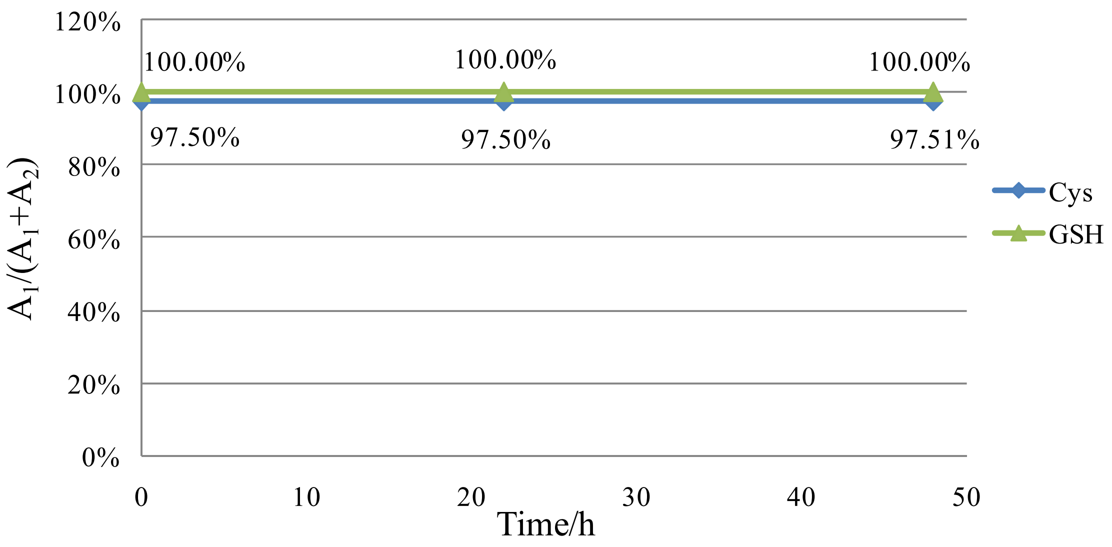

Supplementary Figure S3. Peak intensity for some 5-AIQC-tagged amino analytes as a function of 5-AIQC-to-analyte molar ratios.

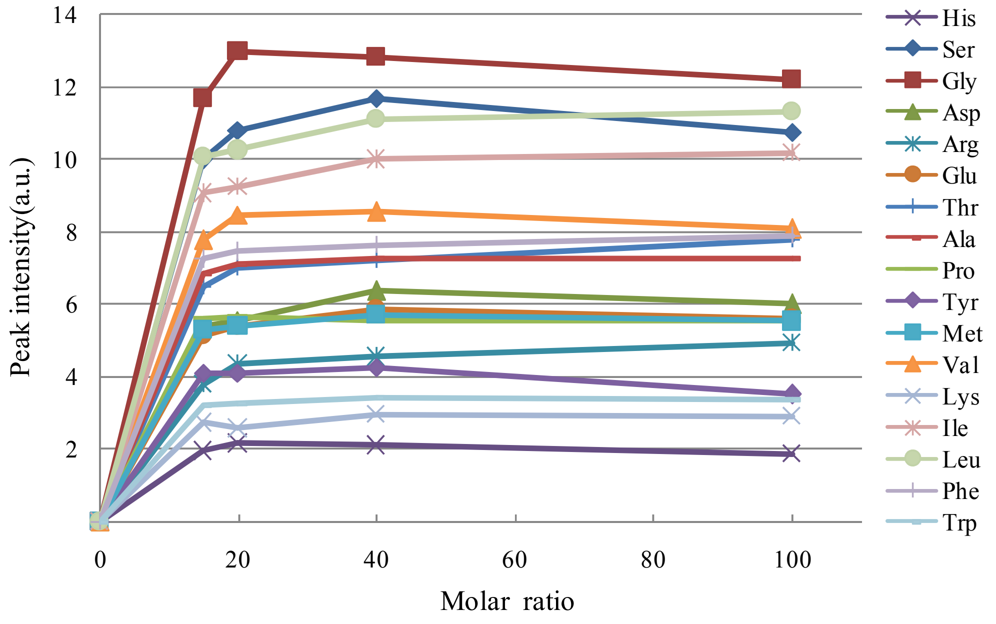

Supplementary Figure S4. UHPLC-MS chromatogram for the 5-AIQC adducts of amino analytes showing twin peaks. (A): DL-2,6-diaminopimelic acid; (B) DL-lanthionine; (C): L-glutathione (reduced form); (D): γ-Glu-Cys and (E): DL-homocysteine.

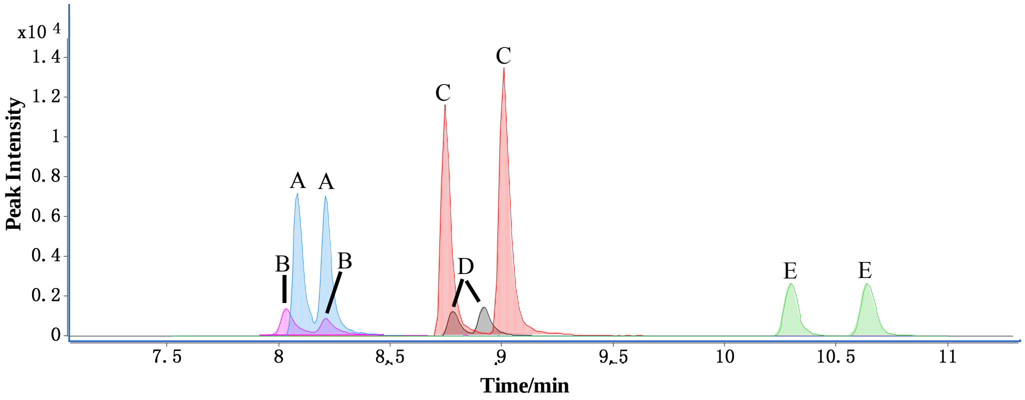

Supplementary Figure S5. UHPLC-MS/MS chromatograms for 5-AQC-tagged 124 amino analytes (keys in Table 1)

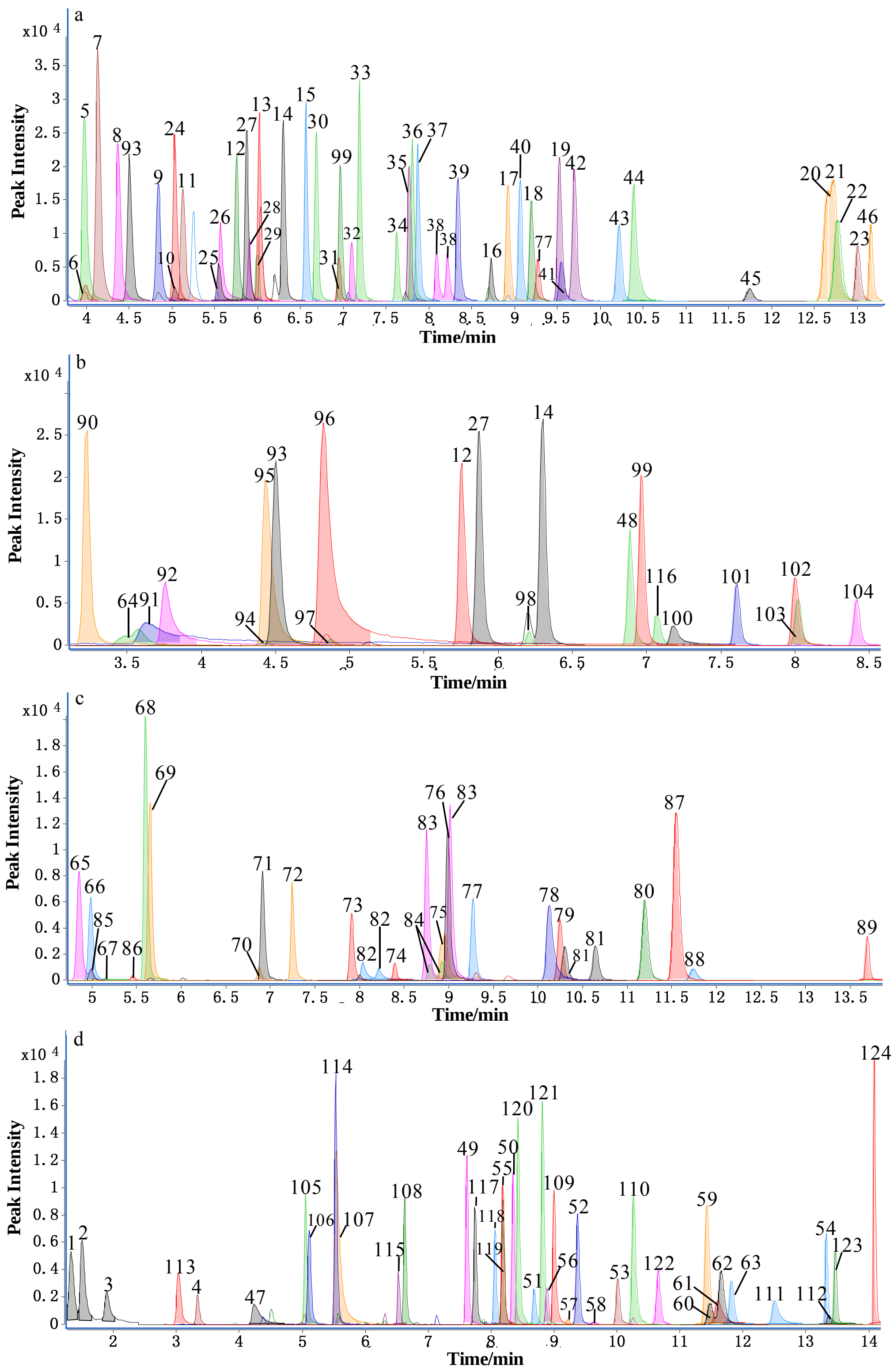

Supplementary Figure S6. UHPLC-MS/MS chromatograms for (A) some small peptides and (B) phenylalanine metabolism and catecholamine biosynthesis .

(A)

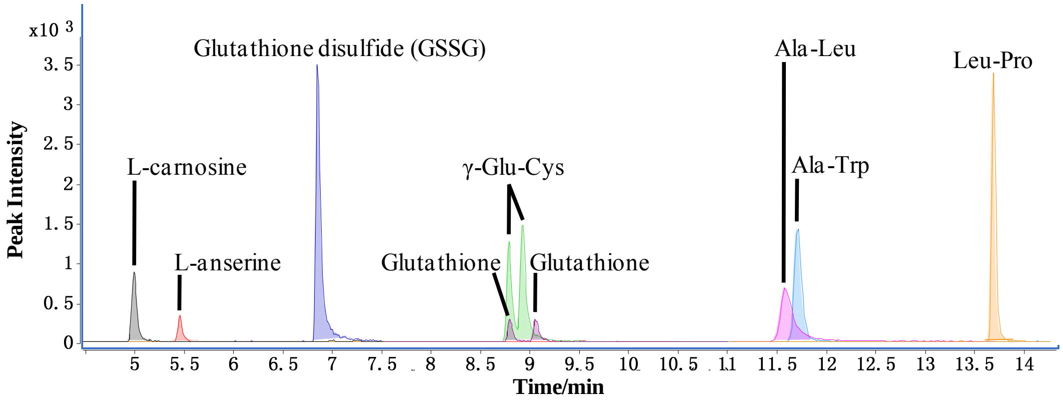

(B)

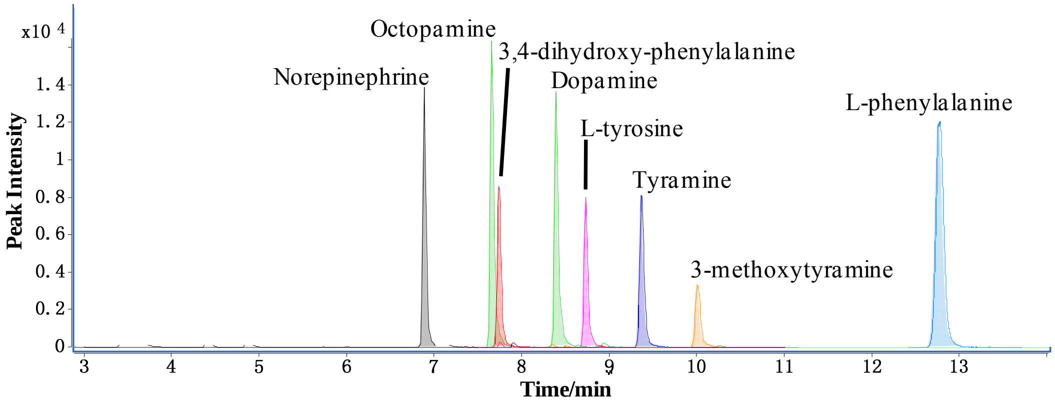

Supplementary Figure S7. pKa measurements for 5-aminoisoquinoline and 6-aminoquinoline (1 mM water solution for both) with ^{1}H NMR method at 298 K. This was done on a Bruker AVANCE III 600 MHz NMR spectrometer as previously described (Xiao, C. et al. *Analyst* **2009**, *134*, 916-925). pH was adjusted using either 0.2 M HCl or 0.2 M NaOH solution to obtain the pH2.5~12. Black squares are experimental values whereas the red lines are fitted data from the following equation:

$$\delta_{obs} = \delta_{min} + \sum_{i=0,n}\left[\Delta_i \Big/ 1 + 10^{(pH - pK_{ai})}\right]$$

where δ_{obs} is the observed chemical shift at a given pH, δ_{min} is chemical shift of a compound in the fully ionized form (when pH is about pKa+2); pK_{ai} and Δ_i denote the apparent ionization constant and the chemical shift difference between fully ionized and unionized forms for the *i*th ionization process, respectively; *n* is the number of ionization processes in a molecule with two ionization processes considered. The calculated pKa values were 5.31±0.07 and 4.95±0.03 for 5-aminoisoquinoline and 6-aminoquinoline, respectively.

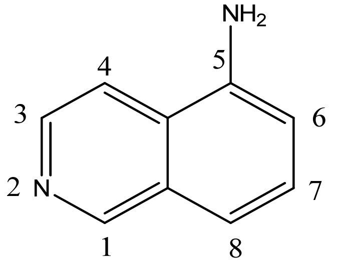

5-aminoisoquinoline

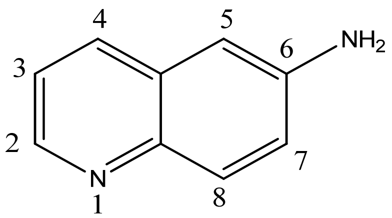

6-aminoquinoline

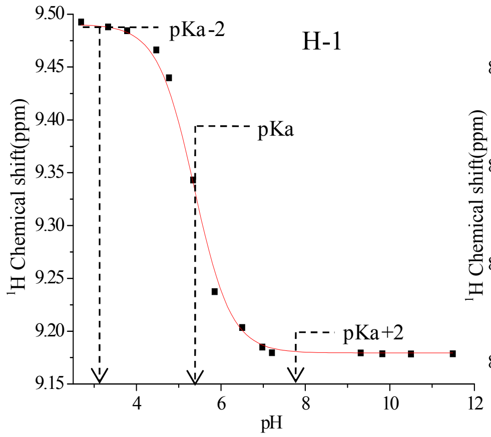

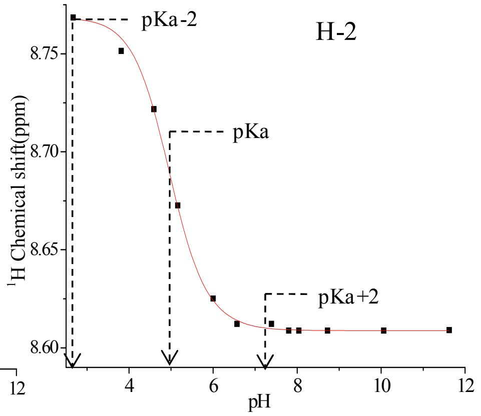

Supplementary Figure S8. Spectroscopic data for 5-AIQC. (a) ^{1}H NMR spectrum in acetonitrile-d_3; (b) QTOF-MS spectrum in positive ion mode (in acetonitrile).

(a)

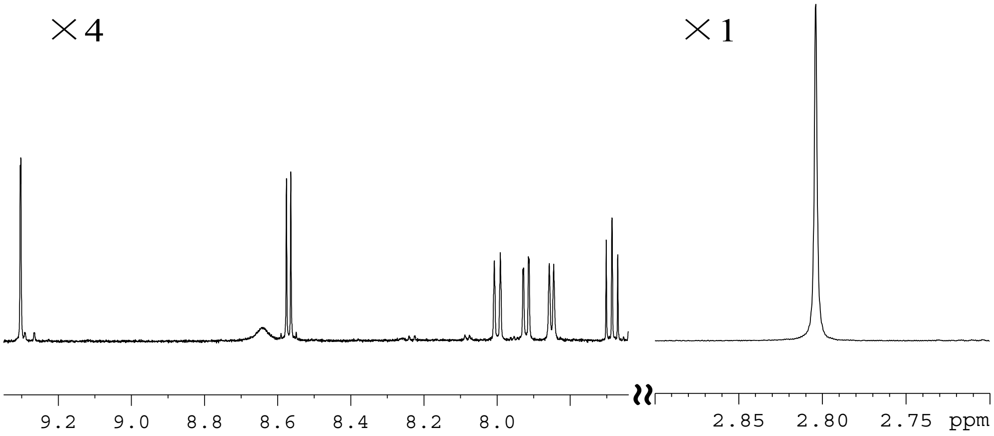

(b)

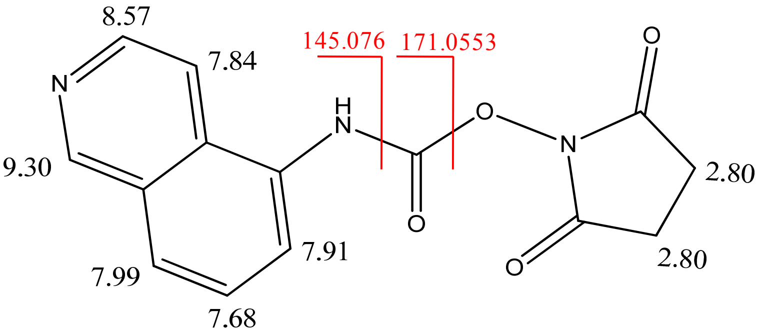

5-AIQC([M+H]$^{+}$:286.0822)

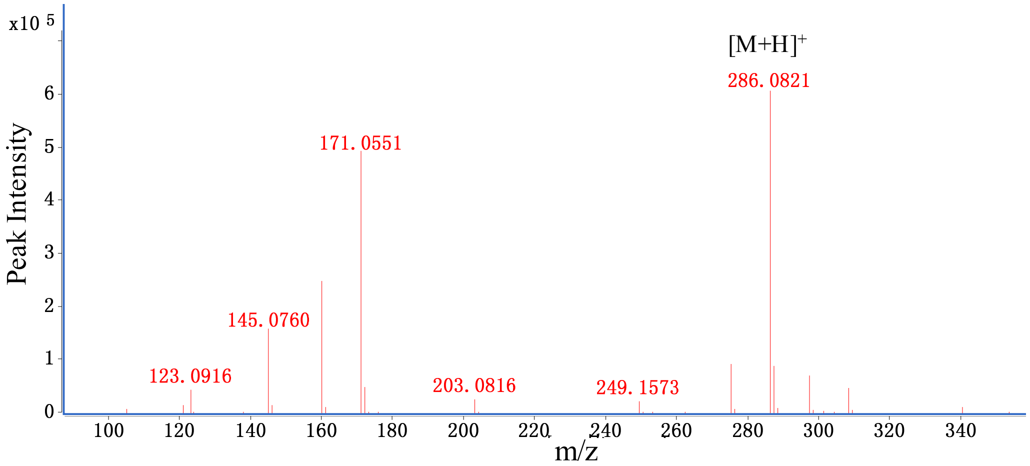

Supplementary Table S1 Solutions of mixed standards (μM) for method validation

	Mix1	Mix2	Mix3	Mix4	Mix5	Mix6	Mix7	Mix8	Mix9
L-Asparagine	200	100	50	25	10	5	1	0.5	0.1
L-Histidine	200	100	50	25	10	5	1	0.5	0.1
L-Serine	200	100	50	25	10	5	1	0.5	0.1
Glycine	200	100	50	25	10	5	1	0.5	0.1
L-Glutamine	200	100	50	25	10	5	1	0.5	0.1
L-Arginine	200	100	50	25	10	5	1	0.5	0.1
L-Aspartic acid	200	100	50	25	10	5	1	0.5	0.1
L-Glutamic acid	200	100	50	25	10	5	1	0.5	0.1
L-Threonine	200	100	50	25	10	5	1	0.5	0.1
L-Alanine	200	100	50	25	10	5	1	0.5	0.1
L-Proline	200	100	50	25	10	5	1	0.5	0.1
L-Tyrosine	100	50	25	12.5	5	2.5	0.5	0.25	0.05
L-Methionine	200	100	50	25	10	5	1	0.5	0.1
L-Lysine	200	100	50	25	10	5	1	0.5	0.1
L-Valine	200	100	50	25	10	5	1	0.5	0.1
L-Isoleucine	200	100	50	25	10	5	1	0.5	0.1
L-Leucine	200	100	50	25	10	5	1	0.5	0.1
DL-Phenylalanine	200	100	50	25	10	5	1	0.5	0.1
L-Tryptophan	200	100	50	25	10	5	1	0.5	0.1
D-Homoserine	200	100	50	25	10	5	1	0.5	0.1
β-alanine	200	100	50	25	10	5	1	0.5	0.1
L-Citrulline	200	100	50	25	10	5	1	0.5	0.1
L-Homoarginine	100	50	25	12.5	5	2.5	0.5	0.25	0.05
γ-Aminobutyric acid	200	100	50	25	10	5	1	0.5	0.1
L-Homocitrulline	200	100	50	25	10	5	1	0.5	0.1
L-2-aminoadipic acid	100	50	25	12.5	5	2.5	0.5	0.25	0.05
DL-3-Aminoisobutyric acid	200	100	50	25	10	5	1	0.5	0.1
2-Aminoisobutyric acid	200	100	50	25	10	5	1	0.5	0.1
5-Aminovaleric acid	200	100	50	25	10	5	1	0.5	0.1
L-2-Aminobutyric acid	200	100	50	25	10	5	1	0.5	0.1
2,4-diaminobutanoic acid	200	100	50	25	10	5	1	0.5	0.1
DL-2,6-Diaminopimelic acid	200	100	50	25	10	5	1	0.5	0.1
L-Ornithine	200	100	50	25	10	5	1	0.5	0.1
6-Aminocaproic acid	200	100	50	25	10	5	1	0.5	0.1
L-Norvaline	200	100	50	25	10	5	1	0.5	0.1
D−(−)-α-Phenylglycine	200	100	50	25	10	5	1	0.5	0.1
L-Kynurenine	100	50	25	12.5	5	2.5	0.5	0.25	0.05
L-Norleucine	100	50	25	12.5	5	2.5	0.5	0.25	0.05
(−)-Norepinephrine	200	100	50	25	10	5	1	0.5	0.1
(±)-Octopamine	200	100	50	25	10	5	1	0.5	0.1
Dopamine	200	100	50	25	10	5	1	0.5	0.1
Tyramine	200	100	50	25	10	5	1	0.5	0.1

3-Methoxytyramine	50	25	12.5	6.25	2.5	1.25	0.25	0.125	0.025
Tryptamine	200	100	50	25	10	5	1	0.5	0.1
4-Aminophenol	200	100	50	25	10	5	1	0.5	0.1
4-Aminohippuric acid	200	100	50	25	10	5	1	0.5	0.1
3-Aminobenzoic acid	200	100	50	25	10	5	1	0.5	0.1
3-Aminosalicylic acid	100	50	25	12.5	5	2.5	0.5	0.25	0.05
4-Aminobenzoic acid	200	100	50	25	10	5	1	0.5	0.1
L-Cysteic acid	200	100	50	25	10	5	1	0.5	0.1
Taurine	200	100	50	25	10	5	1	0.5	0.1
Hypotaurine	100	50	25	12.5	5	2.5	0.5	0.25	0.05
DL-Methionine sulfone	200	100	50	25	10	5	1	0.5	0.1
DL-methionine sulfoxide	200	100	50	25	10	5	1	0.5	0.1
Glutathione disulfide	40	20	10	5	2	1	0.2	0.1	0.02
L-Cystine	50	25	12.5	6.25	2.5	1.25	0.25	0.125	0.025
Cystamine	100	50	25	12.5	5	2.5	0.5	0.25	0.05
L-Homocystine	50	25	12.5	6.25	2.5	1.25	0.25	0.125	0.025
Cystathionine	100	50	25	12.5	5	2.5	0.5	0.25	0.05
S-(2-Aminoethyl)-L-cysteine	200	100	50	25	10	5	1	0.5	0.1
L-Cysteine	100	50	25	12.5	5	2.5	0.5	0.25	0.05
Djenkolic Acid	200	100	50	25	10	5	1	0.5	0.1
Cysteamine	100	50	25	12.5	5	2.5	0.5	0.25	0.05
DL-Ethionine	100	50	25	12.5	5	2.5	0.5	0.25	0.05
DL-Homocysteine	100	50	25	12.5	5	2.5	0.5	0.25	0.05
Glutathione	100	50	25	12.5	5	2.5	0.5	0.25	0.05
L-Carnosine	200	100	50	25	10	5	1	0.5	0.1
Ala-leu	200	100	50	25	10	5	1	0.5	0.1
Ala-Trp	50	25	12.5	6.25	2.5	1.25	0.25	0.125	0.025
trans-4-Hydroxy-L-proline	200	100	50	25	10	5	1	0.5	0.1
O-Phosphorylethanolamine	200	100	50	25	10	5	1	0.5	0.1
Sarcosine	200	100	50	25	10	5	1	0.5	0.1
3-Methyl-L-histidine	100	50	25	12.5	5	2.5	0.5	0.25	0.05
1-Methyl-L-histidine	100	50	25	12.5	5	2.5	0.5	0.25	0.05
Asymmetric dimethylarginine	50	25	12.5	6.25	2.5	1.25	0.25	0.125	0.025
o-acetyl-L-serine	200	100	50	25	10	5	1	0.5	0.1
Nα-Acetyl-L-lysine	200	100	50	25	10	5	1	0.5	0.1
DL-5-Hydroxylysine	100	50	25	12.5	5	2.5	0.5	0.25	0.05
5-Hydroxy-L-tryptophan	200	100	50	25	10	5	1	0.5	0.1
4-Hydroxy-L-isoleucine	50	25	12.5	6.25	2.5	1.25	0.25	0.125	0.025
Ethanolamine	200	100	50	25	10	5	1	0.5	0.1
Methylamine	200	100	50	25	10	5	1	0.5	0.1
Agmatine	200	100	50	25	10	5	1	0.5	0.1
Ethylamine	200	100	50	25	10	5	1	0.5	0.1
Putrescine	200	100	50	25	10	5	1	0.5	0.1
Cadaverine	200	100	50	25	10	5	1	0.5	0.1

Spermine	200	100	50	25	10	5	1	0.5	0.1
Prolinamide	200	100	50	25	10	5	1	0.5	0.1
Allantoin	100	50	25	12.5	5	2.5	0.5	0.25	0.05
5-Hydroxydopamine	50	25	12.5	6.25	2.5	1.25	0.25	0.125	0.025
3,4-dihydroxy-DL-phenylalanine	200	100	50	25	10	5	1	0.5	0.1
DL-Normetanephrine	100	50	25	12.5	5	2.5	0.5	0.25	0.05
1,3-Diaminopropane	200	100	50	25	10	5	1	0.5	0.1
1,2-Diaminopropane	200	100	50	25	10	5	1	0.5	0.1
L-Tryptophanamide	100	50	25	12.5	5	2.5	0.5	0.25	0.05

Supplementary Table S2. Metabolic pathways covered by this quantification method

1. Arg Pro Metabolism	L-Arginine
	L-Proline
	L-Glutamate
	Sarcosine
	L-Ornithine
	trans-4-Hydroxy-L-proline
	Agmatine
	Putrescine
	5-Aminovaleric acid
	gamma-Aminobutyric acid
	1,3-Diaminopropane
	S-Adenosyl-L-methionine
	Spermidine
	Spermine
2. Urea Cycle and Arginine biosynthesis	L-Glutamine
	NH3
	L-Glutamate
	L-Aspartate
	L-Ornithine
	L-Homocitrulline
	L-Citrulline
	L-Argininosuccinate
	L-Arginine
3. Tryptophan metabolism	L-Tryptophan
	5-Hydroxy-L-tryptophan
	Serotonin
	5-Hydroxyindoleacetate
	N-Acetyl-5-hydroxytryptamine
	Tryptamine
	L-Kynurenine
	3-Hydroxy-L-kynurenine
	3-Hydroxyanthranilic acid
4.Phenylalanine Metabolism	L-Tyrosine
	L-Phenylalanine
5. Tyrosine Metabolism	L-Tyrosine
	Tyramine
	3,4-Dihydroxy-L-phenylalanine(L-Dopa）
	Dopamine
	3-Methoxytyramine
	Norepinephrine
	L-Normetanephrine

6. Alanine, aspartate and glutamate metabolism	L-Alanine
	L-Aspartate
	L-Argininosuccinate
	L-Asparagine
	NH3
	L-Glutamate
	gamma-Aminobutyric acid
	L-Glutamine
7. Glutathione Metabolism	Glutathione disulfide
	Glutathione
	L-Glutamate
	L-Cysteine
	Glycine
	gamma-L-Glutamyl-L-cysteine
	L-Ornithine
	Putrescine
	Spermidine
	Spermine
	Cadaverine
8. Taurine & Hypotaurine Metabolism	Taurine
	L-Cysteine
	Hypotaurine
	Cysteamine
	L-Cysteic acid
	L-Alanine
	L-Glutamate
9. Sulfur Metabolism	Taurine
	L-Serine
	L-Cysteine
	L-Homocysteine
	L-Homoserine
10. β-Alanine metabolism	beta-Alanine
	L-Aspartate
	Anserine
	1,3-Diaminopropane
	Spermidine
	Spermine
	4-Aminobutanoate
	L-Histidine
	Carnosine
11. Val Leu Ile Metabolism	L-Threonine
	L-Isoleucine

	L-Valine
	L-Leucine
	L-2-Aminobutyric acid
12.Cysteine and methionine metabolism	L-Serine
	O-Acetyl-L-serine
	L-Cysteine
	O-Phospho-L-serine
	Glutathione
	L-Cystine
	L-Cysteic acid
	L-Alanine
	2-Aminobutanoate
	L-Cystathionine
	L-Aspartate
	L-Homoserine
	L-Serine
	L-Homocysteine
	L-Homocystine
	L-Methionine
	L-Methionine S-oxide
	S-Adenosyl-L-methionine
	S-Adenosyl-L-homocysteine
13. Glycine, serine and threonine metabolism	O-Phospho-L-serine
	L-serine
	L-Tryptophan
	Glycine
	L-Cystathionine
	L-Cysteine
	Sarcosine
	NH3
	L-Threonine
	L-Homoserine
	L-Aspartate
	L-2,4-diaminobutyric acid
14. Lysine degradation	L-Lysine
	L-Homocitrulline
	L-Homoarginine
	Saccharopine
	L-2-Aminoadipate
	5-Hydroxy-L-lysine
	Pipecolic acid
	Nα-Acetyl-L-lysine

	5-Aminovaleric acid
	6-aminocaproic acid
	Cadaverine
15. Histidine metabolism	N6,N6,N6-Trimethyl-L-lysine
	L-Histidine
	1-Methylhistidine
	Anserine
	Carnosine
	Histamine
	L-Aspartate
16. Polyamine pathway	L-Glutamate
	Putrescine
	Spermidine
	Spermine
	Cadaverine
17. Pyrimidine and Purine metabolism	L-Glutamate
	3-Aminoisobutyric acid
	β-Alanine
	Allantoin
18. Drug metabolism	Procaine
	4-Aminohippuric acid
	2-Aminoisobutyric acid
	p-Aminophenol
	Desipramine
19. Gut micriobiota metabolism	Methylamine
	2,6-Diaminopimelic acid
20. Posttranslational modifications	O-Phospho-L-tyrosine
	N6-Acetyl-L-lysine
	O-Phospho-L-threonine
	O-Phospho-L-serine
	Nε,Nε,Nε-Trimethyllysine
21. Glycerophospholipid metabolism	*O*-Phosphorylethanolamine
	Ethanolamine
	Sacosine

Supplementary Table S3. Intra- and inter-day variations (RSD %) for retention time (RT) and concentration (C) of analytes (n=5). RT_L, RT_M, RT_H, C_L, C_M, C_H were parameters at low, intermediate and high concentration range.

		Intra-day (n=5, RSD,%)						Inter-day (n=5, RSD,%)					
**Analytes**	**RT**	**RT_L**	**C_L**	**RT_M**	**C_M**	**RT_H**	**C_H**	**RT_L**	**C_L**	**RT_M**	**C_M**	**RT_H**	**C_H**
L-Asparagine	3.847	3.5	5.1	2.1	7.5	0.6	7.2	4.8	7.3	5.1	11.9	5.9	8.8
L-Histidine	3.957	2.4	2.1	1.8	6.6	0.7	6.0	3.7	9.9	4.3	10.4	5.1	14.8
L-Serine	4.024	3.0	13.7	2.0	8.7	0.5	6.7	4.5	12.6	4.8	13.0	5.7	10.9
Glycine	4.278	2.7	11.4	1.7	7.5	0.6	7.4	4.3	9.9	4.6	13.9	5.4	14.4
L-Glutamine	4.74	2.2	12.2	1.3	7.5	0.4	6.0	3.3	12.7	3.5	12.2	4.1	11.2
L-Arginine	5.016	1.2	2.6	0.9	7.4	0.4	6.7	2.3	2.3	2.6	8.6	3.2	13.9
L-Aspartic acid	5.021	1.6	11.1	1.0	9.2	0.3	8.5	2.9	13.7	3.1	14.2	3.7	12.0
L-Glutamic acid	5.703	0.9	7.0	0.6	10.7	0.2	5.0	2.1	5.8	2.2	12.2	2.8	10.2
L-Threonine	5.979	0.8	13.8	0.5	5.9	0.2	5.9	1.9	12.0	2.0	10.0	2.5	12.9
L-Alanine	6.27	0.7	11.8	0.5	10.9	0.2	5.7	1.8	10.1	1.9	12.2	2.5	12.8
L-Proline	6.538	0.5	11.5	0.4	9.5	0.2	4.3	1.5	9.2	1.7	14.0	2.1	10.2
L-Tyrosine	8.709	0.2	7.0	0.2	7.7	0.1	4.2	1.4	12.9	1.5	11.5	2.0	9.2
L-Methionine	8.911	0.4	7.7	0.3	9.4	0.1	4.5	1.6	6.6	1.9	11.3	2.4	12.2
L-Lysine	9.202	0.8	5.9	0.5	7.1	0.2	7.4	1.9	7.0	2.1	8.8	2.5	10.2
L-Valine	9.515	0.4	12.1	0.3	7.4	0.2	3.7	2.1	10.8	2.3	10.9	2.9	8.9
L-Isoleucine	12.671	0.5	9.5	0.4	8.1	0.3	5.0	1.6	13.7	1.7	11.8	1.7	13.3
L-Leucine	12.738	0.5	9.0	0.3	7.4	0.3	4.8	1.6	12.6	1.7	10.1	1.8	12.3
DL-Phenylalanine	12.783	0.4	11.6	0.4	6.7	0.2	4.9	1.6	14.5	1.7	10.7	2.0	12.3
L-Tryptophan	12.992	0.2	7.6	0.1	5.8	0.1	4.3	1.0	8.5	1.1	13.3	1.3	13.1
D-Homoserine	4.942	1.2	8.3	1.1	5.2	0.4	6.0	3.3	13.5	3.1	13.5	3.7	14.5
β-alanine	5.844	0.8	10.3	0.6	4.4	0.2	5.3	2.0	13.1	2.1	7.6	2.7	10.0
L-Citrulline	5.867	0.8	13.7	0.5	10.9	0.2	4.8	1.8	14.4	1.9	10.9	2.5	13.2
L-Homoarginine	5.934	0.2	9.9	0.4	7.5	0.2	5.3	2.5	14.7	1.6	11.3	2.1	13.3
γ-Aminobutyric acid	6.665	0.6	8.5	0.4	8.9	0.2	5.5	1.6	6.9	1.7	13.6	2.3	9.6
L-Homocitrulline	6.946	0.3	6.5	0.3	5.2	0.2	5.7	1.5	7.2	1.5	13.0	2.0	11.7
L-2-aminoadipic acid	7.075	0.4	14.0	0.3	9.6	0.1	5.5	1.5	12.6	1.6	15.1	2.1	11.9
3-aminoisobutyric acid	7.172	0.5	11.5	0.3	12.1	0.2	3.9	1.6	9.4	1.8	10.4	2.3	8.5
2-aminoisobutyric acid	7.613	0.4	54.0	0.3	53.0	0.2	40.7	1.3	82.3	1.7	90.6	2.2	78.6
5-aminovaleric acid	7.754	0.3	5.5	0.3	5.6	0.1	4.1	1.6	5.4	1.7	9.2	2.1	9.7
L-2-aminobutyric acid	7.792	0.4	11.4	0.3	8.9	0.2	3.9	1.6	10.0	1.7	13.3	2.2	11.5
2,4-diaminobutyric acid	7.859	0.5	6.1	0.5	7.2	0.2	6.4	1.7	5.5	1.7	11.5	2.1	11.5
2,6-diaminopimelic acid	8.195	0.7	8.3	0.8	8.3	0.9	7.5	1.5	9.9	1.9	11.9	1.9	11.2
L-Ornithine	8.321	0.6	10.0	0.4	8.1	0.2	7.5	1.5	9.7	1.6	12.5	2.0	11.9
6-Aminocaproic acid	9.053	0.3	8.5	0.3	5.3	0.2	5.6	1.8	8.9	1.9	12.9	2.4	8.3
L-Norvaline	9.709	0.3	7.1	0.3	7.4	0.2	4.5	2.3	7.4	2.3	10.2	2.9	11.0
D−(−)-α-phenylglycine	10.224	0.3	5.4	0.2	9.4	0.1	5.6	2.2	7.6	2.4	15.4	3.0	13.6
L-Kynurenine	11.753	0.4	4.8	0.4	10.0	0.2	5.6	2.7	5.5	2.8	13.9	3.6	13.9
L-Norleucine	13.156	0.2	8.7	0.1	7.3	0.0	4.7	0.9	7.4	0.8	15.5	1.2	13.3
(−)-Norepinephrine	6.882	0.6	6.5	0.4	7.8	0.2	4.8	1.5	4.9	1.7	13.3	2.1	12.5

(±)-Octopamine	7.59	0.4	4.1	0.3	8.0	0.2	3.9	1.6	6.1	1.7	12.0	2.1	9.5
Dopamine	8.336	0.4	4.7	0.3	10.4	0.2	3.9	1.4	4.6	1.6	14.2	2.1	12.6
Tyramine	9.358	0.4	8.0	0.3	9.3	0.2	3.9	2.0	7.3	2.1	10.7	2.7	7.5
3-Methoxytyramine	10.008	0.6	12.2	0.4	8.1	0.2	4.1	2.0	10.1	2.3	11.7	2.9	8.4
Tryptamine	13.305	0.1	2.7	0.1	6.9	0.1	5.2	0.8	2.7	0.8	13.9	1.0	13.0
4-Aminophenol	8.165	0.4	3.9	0.4	5.3	0.2	4.0	1.8	3.6	1.8	10.6	2.3	11.6
4-Aminohippuric acid	9.202	0.2	13.9	0.5	7.8	0.2	6.5	3.0	19.7	2.0	12.0	2.6	7.9
3-Aminobenzoic acid	11.477	0.7	3.7	0.8	9.0	0.3	6.8	3.5	5.0	3.6	13.3	4.5	10.3
3-Aminosalicylic acid	11.574	0.3	4.3	0.2	8.1	0.1	4.9	2.5	4.5	2.5	14.0	3.4	17.8
4-Aminobenzoic acid	11.649	0.6	4.8	0.5	6.4	0.3	5.1	3.1	4.3	3.3	8.9	4.1	6.3
L-Cysteic acid	3.473	3.2	10.3	3.1	7.2	0.9	7.8	6.6	14.7	6.4	21.3	6.8	18.3
Taurine	4.733	2.4	13.8	1.4	6.2	0.4	6.0	3.5	12.7	3.7	12.7	4.3	13.4
Hypotaurine	4.875	2.2	6.5	1.2	4.8	0.4	7.1	3.3	8.4	3.4	13.0	4.0	12.8
Methionine sulfone	5.546	0.8	6.1	0.7	9.6	0.2	4.9	2.4	9.6	2.3	13.6	2.9	14.0
Methionine sulfoxide	5.606	0.7	11.1	0.7	9.7	0.2	6.7	2.2	11.0	2.1	11.7	2.7	14.7
Glutathione disulfide	6.844	0.5	13.9	0.3	14.0	0.1	5.3	1.3	14.0	1.6	12.7	2.1	13.7
L-Cystine	6.882	0.6	9.9	0.4	10.6	0.1	5.9	1.8	9.3	1.9	16.3	2.5	16.7
Cystamine	7.232	0.7	9.7	0.4	8.4	0.2	5.1	1.7	9.3	2.0	14.4	2.2	14.0
L-Homocystine	7.896	0.5	9.1	0.3	9.8	0.1	4.8	1.6	9.6	1.8	11.5	2.3	13.0
Cystathionine	8.956	0.6	11.1	0.3	9.1	0.2	9.0	1.5	12.2	1.6	15.0	2.1	14.0
S-(2-aminoethyl)-L-Cys	8.985	0.5	3.5	0.4	7.4	0.2	8.7	1.9	3.9	1.9	13.0	2.3	12.4
L-Cysteine	9.261	0.5	7.9	0.2	11.2	0.1	6.1	1.8	9.5	1.9	11.6	2.4	12.5
Djenkolic Acid	10.112	0.4	5.2	0.4	9.9	0.2	6.9	2.4	7.1	2.3	13.7	2.9	14.5
Cysteamine	10.246	0.7	5.1	0.5	6.4	0.2	3.2	2.3	4.6	2.5	9.3	3.1	7.8
DL-Ethionine	11.201	0.4	7.2	0.4	9.6	0.2	5.3	2.8	8.7	3.0	14.5	3.6	12.4
DL-Homocysteine	10.291	1.4	9.4	1.8	6.3	1.3	3.9	1.3	7.9	3.1	10.5	4.0	10.1
	10.611												
Glutathione	8.784	1.7	10.0	1.5	11.9	0.1	4.9	1.9	13.9	2.2	13.1	1.6	13.6
	9.053												
L-Carnosine	4.964	0.7	3.7	1.0	7.9	0.4	5.6	3.0	3.1	2.7	12.2	3.3	13.9
Ala-leu	11.574	0.7	8.4	0.5	8.0	0.2	3.6	2.7	8.0	3.3	7.8	3.8	7.9
Ala-Trp	11.753	0.4	5.0	0.4	7.4	0.2	4.5	2.6	5.1	2.8	13.4	3.6	12.4
trans-4-hydroxy-L-Pro	3.091	4.9	4.0	3.0	9.6	0.8	6.2	6.0	11.9	6.4	14.2	6.8	11.9
O-PE	3.875	2.1	5.9	2.1	5.5	0.6	6.3	5.1	5.2	5.0	10.6	5.8	9.9
Sarcosine	4.412	2.4	53.9	1.7	27.2	0.5	45.6	5.7	72.5	3.9	78.4	4.8	85.2
3-Methyl-L-histidine	4.457	3.1	3.9	1.2	12.5	0.4	7.0	3.8	14.4	2.7	12.2	3.4	13.1
1-Methyl-L-histidine	4.897	0.4	14.2	1.3	11.6	0.6	5.5	5.3	12.6	3.7	14.5	4.5	12.2
ADMA	6.24	0.7	7.4	0.5	8.2	0.2	4.7	1.9	13.5	1.6	13.5	2.6	10.7
O-acetyl-L-serine	6.941	0.3	7.3	0.3	7.1	0.1	5.8	1.7	10.3	1.7	11.7	2.3	13.7
Nα-Acetyl-L-lysine	7.59	0.3	8.4	0.2	11.5	0.1	3.6	1.5	8.2	1.5	11.0	2.0	9.3
DL-5-Hydroxylysine	7.986	0.6	13.9	0.4	6.8	0.2	8.1	1.4	12.7	1.6	12.4	2.0	12.8
5-hydroxy-L-Trp	8.001	0.2	2.4	0.2	4.8	0.1	4.9	1.3	4.9	1.4	11.7	1.8	20.3
4-hydroxy-L-Ile	8.404	0.2	18.8	0.2	16.1	0.2	11.5	1.6	19.4	1.6	19.3	2.1	11.8
Ethanolamine	5.024	1.6	12.8	1.0	5.6	0.4	8.4	2.7	11.2	2.9	10.7	3.6	12.5

Methylamine	5.076	1.1	13.4	1.0	5.9	0.5	8.7	3.0	11.5	2.9	14.4	3.6	12.9
Agmatine	5.68	0.4	13.2	0.4	10.9	0.3	4.7	1.6	11.9	1.6	15.9	2.2	10.2
Ethylamine	6.613	0.5	4.6	0.5	9.4	0.2	5.6	2.0	4.4	2.0	13.0	2.5	10.0
Putrescine	9.008	0.6	5.9	0.5	7.0	0.2	8.2	2.1	5.5	2.1	12.2	2.6	10.1
Cadaverine	10.284	1.1	5.0	0.8	5.2	0.4	6.6	2.5	6.6	3.0	9.8	3.6	9.5
Spermine	13.292	0.2	2.3	0.1	6.9	0.1	9.1	0.7	3.7	0.6	9.4	0.7	12.1
Prolinamide	5.501	0.8	12.8	0.6	9.5	0.2	6.3	2.1	10.9	2.0	14.7	2.6	10.7
Allantoin	6.521	0.7	9.3	0.6	16.6	0.2	10.3	1.9	14.9	1.9	15.6	2.4	15.0
5-Hydroxydopamine	7.061	0.4	6.5	0.3	9.3	0.2	4.7	1.7	5.8	1.6	13.1	2.1	12.0
3,4-dihydroxy-DL-Phe	7.725	0.2	5.0	0.2	6.4	0.1	3.9	1.3	4.5	1.4	14.1	1.9	17.2
DL-Normetanephrine	8.045	0.4	9.2	0.3	9.8	0.1	4.7	1.4	7.8	1.6	12.3	2.0	9.7
1,3-Diaminopropane	8.418	0.6	3.5	0.5	5.3	0.2	7.3	1.8	3.3	1.9	8.6	2.3	9.8
1,2-Diaminopropane	8.821	0.6	7.9	0.5	6.7	0.2	8.3	2.0	7.6	2.0	9.3	2.5	13.2
L-Tryptophanamide	10.649	0.5	12.9	0.5	8.4	0.3	2.8	2.7	11.5	2.8	14.4	3.4	13.5

O-PE: O-phosphorylethanolamine; ADMA: asymmetric dimethylarginine; Phe: phenylalanine; Pro: proline; Trp: tryptophan; Ile: isoleucine; Cys: cysteine.

Supplementary Table S4. Sensitivity enhancement by jointly using the Stream Jet ion souses and iFunnel technology (on an Agilent 6495 triple-quad spectrometer)

Name	Neutral formula	Theoretical (m/z)	Q1 (m/z)	Q3 (m/z)	CE	RT	LOD (fmol)	Linear range (μM)	R^2
L-Asparagine	$C_{14}H_{14}N_4O_4$	303.1088	303	171	20	3.999	0.2	0.1-200	0.9983
L-Histidine	$C_{16}H_{15}N_5O_3$	326.1248	326	171	20	4.161	1	0.3-200	0.996
L-Serine	$C_{13}H_{13}N_3O_4$	276.0979	276	171	20	4.157	0.2	0.1-200	0.9941
Glycine	$C_{12}H_{11}N_3O_3$	246.0873	246	171	20	4.407	0.2	0.1-200	0.9945
L-Glutamine	$C_{15}H_{16}N_4O_4$	317.1244	317	171	20	4.905	0.4	0.1-200	0.995
L-Arginine	$C_{16}H_{20}N_6O_3$	345.167	345	171	20	5.169	3.9	1.3-200	0.9953
L-Aspartic acid	$C_{14}H_{13}N_3O_5$	304.0928	304	171	20	5.172	0.5	0.2-200	0.9925
L-Glutamic acid	$C_{15}H_{15}N_3O_5$	318.1084	318	171	20	5.831	0.2	0.1-200	0.9956
L-Threonine	$C_{14}H_{15}N_3O_4$	290.1135	290	171	20	6.092	0.1	0.04-200	0.996
L-Alanine	$C_{13}H_{13}N_3O_3$	260.103	260	171	20	6.384	0.1	0.05-200	0.9984
L-Proline	$C_{15}H_{15}N_3O_3$	286.1186	286	171	20	6.609	0.1	0.05-200	0.9994
L-Tyrosine	$C_{19}H_{17}N_3O_4$	352.1292	352	171	20	8.902	0.2	0.1-100	0.9924
L-Methionine	$C_{15}H_{17}N_3O_3S$	320.1063	320	171	20	9.032	0.3	0.1-200	0.9949
L-Lysine	$C_{26}H_{26}N_6O_4$	244.1081	244	171	10	9.335	0.3	0.1-200	0.991
L-Valine	$C_{15}H_{17}N_3O_3$	288.1343	288	171	20	9.635	0.3	0.1-200	0.9955
L-Isoleucine	$C_{16}H_{19}N_3O_3$	302.1499	302	171	20	12.879	0.4	0.1-200	0.9912
L-Leucine	$C_{16}H_{19}N_3O_3$	302.1499	302	171	20	12.947	0.3	0.1-200	0.9938
DL-Phenylalanine	$C_{19}H_{17}N_3O_3$	336.1343	336	171	20	13.039	0.3	0.1-200	0.9952
L-Tryptophan	$C_{21}H_{18}N_4O_3$	375.1452	375	171	20	13.268	0.4	0.1-200	0.9945
D-Homoserine	$C_{14}H_{15}N_3O_4$	290.1135	290	171	20	5.073	0.4	0.1-200	0.9976
β-alanine	$C_{13}H_{13}N_3O_3$	260.103	260	171	20	5.965	0.2	0.1-200	0.9949
L-Citrulline	$C_{16}H_{19}N_5O_4$	346.151	346	171	20	5.991	0.7	0.2-200	0.9938
L-Homoarginine	$C_{17}H_{22}N_6O_3$	359.1826	359	171	20	6.049	2.1	0.7-100	0.9982
γ-Aminobutyric acid	$C_{14}H_{15}N_3O_3$	274.1186	274	171	20	6.775	0.3	0.1-200	0.9902

L-Homocitrulline	$C_{17}H_{21}N_5O_4$	360.1666	360	171	20	7.075	0.3	0.1-200	0.9956
L-2-aminoadipic acid	$C_{16}H_{17}N_3O_5$	332.1241	332	171	20	7.198	0.3	0.1-100	0.9914
DL-3-Aminoisobutyric acid	$C_{14}H_{15}N_3O_3$	274.1186	274	171	20	7.282	0.2	0.1-200	0.9909
2-Aminoisobutyric acid	$C_{14}H_{15}N_3O_3$	274.1186	274	171	20	7.744	0.9	0.3-200	0.9981
5-Aminovaleric acid	$C_{15}H_{17}N_3O_3$	288.1343	288	171	20	7.875	0.4	0.1-200	0.9976
L-2-Aminobutyric acid	$C_{14}H_{15}N_3O_2$	274.1186	274	171	20	7.897	0.3	0.1-200	0.9911
2,4-diaminobutanoic acid	$C_{24}H_{22}N_6O_4$	230.0924	230	171	10	7.984	0.8	0.3-200	0.9985
DL-2,6-Diaminopimelic acid	$C_{27}H_{26}N_6O_6$	266.103	266	171	20	8.215/8.343	0.9	0.3-200	0.997
L-Ornithine	$C_{25}H_{24}N_6O_4$	237.1002	237	171	10	8.444	0.4	0.1-200	0.9924
6-Aminocaproic acid	$C_{16}H_{19}N_3O_3$	302.1499	302	171	10	9.194	0.4	0.1-200	0.9959
L-Norvaline	$C_{15}H_{17}N_3O_3$	288.1343	288	171	20	9.833	0.3	0.1-200	0.9936
D−(−)-α-Phenylglycine	$C_{18}H_{15}N_3O_3$	322.1186	322	171	20	10.375	0.6	0.2-200	0.9981
L-Kynurenine	$C_{20}H_{18}N_4O_4$	379.1401	379	171	20	12.044	1.1	0.4-100	0.9982
L-Norleucine	$C_{16}H_{19}N_3O_3$	302.1499	302	171	20	13.325	0.2	0.1-100	0.9953
(−)-Norepinephrine	$C_{18}H_{17}N_3O_4$	340.1292	340	171	20	7.022	0.9	0.3-200	0.9925
(±)-Octopamine	$C_{18}H_{17}N_3O_3$	324.1343	324	171	20	7.723	0.2	0.1-200	0.9968
Dopamine	$C_{18}H_{17}N_3O_3$	324.1343	324	171	20	8.473	0.4	0.1-200	0.9955
Tyramine	$C_{18}H_{17}N_3O_2$	308.1394	308	171	30	9.506	0.3	0.1-200	0.9969
3-Methoxytyramine	$C_{19}H_{19}N_3O_3$	338.1499	338	171	30	10.141	0.2	0.1-50	0.9924
Tryptamine	$C_{20}H_{18}N_4O$	331.1553	331	171	20	13.375	0.7	0.2-200	0.9968
4-Aminophenol	$C_{16}H_{13}N_3O_2$	280.1081	280	171	20	8.311	0.5	0.2-200	0.9987
4-Aminohippuric acid	$C_{19}H_{16}N_4O_4$	365.1244	365	171	30	9.394	4.5	1.5-200	0.9978
3-Aminobenzoic acid	$C_{17}H_{13}N_3O_3$	308.103	308	171	20	11.803	2.5	0.8-200	0.9903
3-Aminosalicylic acid	$C_{17}H_{13}N_3O_4$	324.0979	324	171	20	11.910	3.6	1.2-100	0.9909
4-Aminobenzoic acid	$C_{17}H_{13}N_3O_3$	308.103	308	171	20	11.972	0.8	0.3-200	0.9975
L-Cysteic acid	$C_{13}H_{13}N_3O_6S$	340.0598	340	171	20	3.546	1.7	0.6-200	0.9922

Taurine	$C_{12}H_{13}N_3O_4S$	296.07	296	171	20	4.88	1.2	0.4-200	0.9941
Hypotaurine	$C_{12}H_{13}N_3O_3S$	280.075	280	171	20	5.015	0.5	0.2-100	0.9974
DL-Methionine sulfone	$C_{15}H_{17}N_3O_6S$	352.0962	352	171	40	5.664	0.4	0.1-200	0.9921
DL-methionine sulfoxide	$C_{15}H_{17}N_3O_4S$	336.1013	336	171	20	5.713	0.5	0.1-200	0.9962
Glutathione disulfide	$C_{20}H_{23}N_5O_7S$	478.1391	478	171	30	6.95	0.8	0.3-40	0.9918
L-Cystine	$C_{13}H_{14}N_3O_3S$	292.075	292	171	20	7.003	0.3	0.1-50	0.9905
Cystamine	$C_{12}H_{13}N_3OS$	248.0852	248	171	20	7.329	1.3	0.4-100	0.9954
L-Homocystine	$C_{14}H_{15}N_3O_3S$	306.0907	306	171	20	8.019	0.5	0.2-50	0.9964
Cystathionine	$C_{27}H_{26}N_6O_6S$	282.089	282	171	10	9.099	1.4	0.5-100	0.9922
S-(2-Aminoethyl)-L-Cys	$C_{25}H_{24}N_6O_4S$	253.0863	253	171	10	9.127	0.5	0.2-200	0.9986
L-Cysteine	$C_{19}H_{20}N_4O_5S$	417.1227	417	171	30	9.705	0.9	0.3-100	0.9913
Djenkolic Acid	$C_{27}H_{26}N_6O_6S_2$	298.075	298	171	10	10.342	0.8	0.3-200	0.9948
Cysteamine	$C_{18}H_{20}N_4O_3S$	373.1329	373	171	40	10.385	0.6	0.2-100	0.9947
DL-Ethionine	$C_{16}H_{19}N_3O_3S$	334.122	334	171	20	11.379	0.7	0.2-100	0.9958
DL-Homocysteine	$C_{20}H_{22}N_4O_5S$	431.1384	431	171	30	10.447/10.804	1.7	0.6-100	0.9947
Glutathione	$C_{26}H_{30}N_6O_9S$	603.1868	603	171	40	8.891/9.197	1.7	0.6-100	0.9907
L-Carnosine	$C_{19}H_{20}N_6O_4$	397.1619	397	171	40	5.113	6.8	2.3-200	0.995
Ala-leu	$C_{19}H_{24}N_4O_4$	373.187	373	171	30	11.725	0.1	0.03-200	0.9919
Ala-Trp	$C_{24}H_{23}N_5O_4$	446.1823	446	171	30	11.992	0.3	0.1-50	0.9962
trans-4-Hydroxy-L-Pro	$C_{15}H_{15}N_3O_4$	302.1135	302	171	20	3.192	0.4	0.1-200	0.9993
O-Phosphorylethanolamine	$C_{12}H_{14}N_3O_5P$	312.0744	312	171	20	4.027	0.7	0.2-200	0.9927
Sarcosine	$C_{13}H_{13}N_3O_3$	260.103	260	171	20	4.5	0.5	0.2-200	0.9948
3-Methyl-L-histidine	$C_{17}H_{17}N_5O_3$	340.1404	340	171	20	4.627	2	0.7-100	0.9966
1-Methyl-L-histidine	$C_{17}H_{17}N_5O_3$	340.1404	340	171	20	5.028	3.8	1.3-100	0.9911
ADMA	$C_{18}H_{24}N_6O_3$	373.1983	373	171	30	6.343	12	4.0-50	0.9945
o-acetyl-L-serine	$C_{15}H_{15}N_3O_5$	318.1084	318	171	20	7.052	0.4	0.1-200	0.9935

Nα-Acetyl-L-lysine	$C_{18}H_{22}N_4O_4$	359.1714	359	171	40	7.692	0.3	0.1-200	0.9937
DL-5-Hydroxylysine	$C_{26}H_{26}N_6O_5$	252.1055	252	171	10	8.1	0.3	0.1-100	0.9984
5-Hydroxy-L-tryptophan	$C_{21}H_{18}N_4O_4$	391.1401	391	171	20	8.175	0.3	0.1-200	0.9985
4-Hydroxy-L-isoleucine	$C_{16}H_{19}N_3O_4$	318.1448	318	171	20	8.527	1.8	0.6-50	0.9955
Ethanolamine	$C_{12}H_{13}N_3O_2$	232.1081	232	171	20	5.16	0.1	0.04-200	0.9945
Methylamine	$C_{11}H_{11}N_3O$	202.0975	202	171	40	5.183	0.2	0.1-200	0.9926
Agmatine	$C_{15}H_{20}N_6O$	301.1771	301	171	20	5.764	0.9	0.3-200	0.9947
Ethylamine	$C_{12}H_{13}N_3O$	216.1131	216	171	20	6.687	0.3	0.1-200	0.9937
Putrescine	$C_{24}H_{24}N_6O_2$	215.1053	215	171	20	9.12	0.4	0.1-200	0.9971
Cadaverine	$C_{25}H_{26}N_6O_2$	222.1131	222	171	10	10.395	0.3	0.1-200	0.9906
Spermine	$C_{50}H_{50}N_{12}O_4$	442.2112	442	171	30	13.323	2.3	0.8-200	0.9927
Prolinamide	$C_{15}H_{16}N_4O_2$	285.1346	285	171	20	5.571	0.2	0.1-200	0.9935
Allantoin	$C_{14}H_{12}N_6O_4$	329.0993	329	171	20	6.63	3.8	1.3-100	0.9938
5-Hydroxydopamine	$C_{18}H_{17}N_3O_4$	340.1292	340	171	20	7.168	1	0.3-50	0.9929
3,4-dihydroxy-DL-Phe	$C_{19}H_{17}N_3O_5$	368.1241	368	171	20	7.891	0.8	0.3-200	0.998
DL-Normetanephrine	$C_{19}H_{19}N_3O_4$	354.1448	354	171	30	8.174	0.5	0.2-100	0.9971
1,3-Diaminopropane	$C_{23}H_{22}N_6O_2$	208.0975	208	171	10	8.528	0.4	0.1-200	0.9986
1,2-Diaminopropane	$C_{23}H_{22}N_6O_2$	208.0975	208	171	10	8.911	0.3	0.1-200	0.994
L-Tryptophanamide	$C_{21}H_{19}N_5O_2$	374.1612	374	171	30	10.856	0.5	0.2-100	0.993
